# Dynamics and Sensitivity of the Lifecycle of Hepatitis B Virus

**DOI:** 10.3390/pathogens15020172

**Published:** 2026-02-05

**Authors:** Dmitry Grebennikov, Igor Sazonov, Rostislav Savinkov, Matvey Zakharov, Mark Sorokin, Yakov Mokin, Andreas Meyerhans, Gennady Bocharov

**Affiliations:** 1 Marchuk Institute of Numerical Mathematics of the RAS (INM RAS), 119333 Moscow, Russia; grebennikov_d_s@staff.sechenov.ru (D.G.); r.savinkov@inm.ras.ru (R.S.); g.bocharov@inm.ras.ru (G.B.); 2 Moscow Center of Fundamental and Applied Mathematics at INM RAS, 119333 Moscow, Russia; 3 Institute for Computer Science and Mathematical Modelling, Sechenov First Moscow State Medical University, 119991 Moscow, Russia; mattew.zakharov.03@mail.ru (M.Z.); smark04@inbox.ru (M.S.); 4 Faculty of Science and Engineering, Swansea University, Bay Campus, Fabian Way, Swansea SA1 8EN, UK; 5 Laboratory of Structural Dynamics, Stability and Folding of Proteins, Institute of Cytology, Russian Academy of Sciences, 194064 St. Petersburg, Russia; mokinyakov@mail.ru; 6 ICREA, Pg. Lluis Companys 23, 08010 Barcelona, Spain; andreas.meyerhans@upf.edu; 7 Infection Biology Laboratory, Universitat Pompeu Fabra, 08003 Barcelona, Spain

**Keywords:** HBV, intracellular lifecycle, mathematical model, stochastic description, sensitivity analysis, antivirals

## Abstract

A detailed mathematical model has been developed for the dynamics of hepatitis B virus (HBV) infection in a single cell. It provides a platform for a better quantitative understanding of the biochemical kinetics of the HBV lifecycle. The model is used to study the sensitivity of virus growth, providing a clear ranking of intracellular virus replication processes with respect to their contribution to net viral production. The stochastic formulation of the model enables the quantification of the variability characteristics in viral production, the probability of productive infection and the secretion of protein- and genome-deficient viral particles. An essential difference in infection efficiency between deterministic and stochastic models has been revealed. For example, in the case of MOI=1, the mean value of the total number of mature virions released during the lifecycle of the infection in the stochastic model is 1.06, whereas, in the deterministic model, its value is less than one thousandth and thus close to 0. The model is also used to quantitatively predict the effect of combinations of direct-acting antivirals, such as small interfering RNAs, capsid inhibitors and nucleoside analogues. The model shows that the inhibitory effect of siRNA on viral production is approximately two orders of magnitude higher than that of nucleoside analogues and capsid inhibitors.

## 1. Introduction

Hepatitis B virus (HBV) infection remains a global health problem, with several hundred million chronically infected individuals worldwide [1]. The productive infection of hepatocytes with HBV is based on the formation of the transcriptional template, i.e., covalently closed circularized DNA (cccDNA), in the nucleus [2]. In chronic HBV infection, it is responsible for viral persistence and presents a key obstacle for a cure [3].

Currently, the endpoint for therapy of chronic HBV infection is a functional cure. This is a physiological state that is characterized by the loss of HBsAg, undetectable serum levels of viral DNA and persistence of inactive cccDNA [4]. This is associated with sustained immune-mediated control of hepatitis B virus without the need for further medication [5]. In this regard, a combination therapy, based on the use of anti-HBV drugs and immune modulatory treatments, is considered to be a promising approach to achieve a functional cure. A broad spectrum of potent direct-acting and host-immune system-targeting drugs are available for approved clinical use or clinical trials [6,7]. However, a rational elaboration of the effective regimes of combination therapies (e.g., simultaneous, sequential or add-on) still remains a problem [4,8,9,10].

A number of critical steps in HBV entry, cccDNA maintenance/stability and HBV transcription remain largely unclarified [11]. Mathematical modeling could provide a platform for a better quantitative understanding of the biochemical kinetics of the HBV lifecycle with translational implications for better targeting of HBV infection at a single-cell level.

So far, relatively few mathematical models have been developed to address various aspects of HBV replication. The models presented in [12,13] detail the mechanisms underlying two patterns of HBV replication, referred to as arrested and explosive replication. The focus has been on the recycling of the HBV virions as a positive regulatory feedback driving explosive replication. The mechanisms underlying the accumulation of cccDNA in the nucleus of hepatocytes have been explored via a combination of mathematical modeling and in vitro studies of HBV infection and treatment [1]. The formulated model considers the dynamics of three characteristic elements of infection, i.e., cccDNA, intracellular HBV and extracellular HBV. A multiscale model has been developed to study the action of capsid assembly modulators on HBV DNA and RNA levels in [14]. The intracellular lifecycle of HBV is limited to the dynamics of encapsidated pregenomic DNA (pgDNA) and relaxed circular DNA (rcDNA) only. A similar simple description of the intracellular dynamics of HBV replication via intracellular DNA, RNA and encapsidated pgRNA is presented in [15]. The same type of intracellular replication dynamics is covered in [16]. A more detailed consideration of intracellular HBV replication can be found in [17]. The authors consider the dynamics of infecting rcDNA, cccDNA, single-stranded DNA (ssDNA), and double-stranded DNA (dsDNA) within an infected cell and study the emergence of refractory cells by immune-mediated clearance. The most elaborated mathematical model developed so far is presented in [18]. It is formulated using a stochastic Gillespie approach with a detailed description of (i) the sequential formation of the encapsidation of the RNP complex and (ii) HBV replication (formation of cccDNA, its transcription, viral protein synthesis, rcDNA-containing nucleocapsid formation, and secretion of complete and incomplete particles). The model examines the effects of various antiviral drugs. Its distinguished feature is a detailed description of the encapsidation and SVP formation in the process of viral particles production.

The features of the existing models are summarized in Table 1.

However, the existing models do not account for some biochemical reactions involved in the infection cycle in a cell. Therefore, we develop here a novel mathematical model based on a more detailed biochemical scheme that considers all the important biochemical reactions. In addition, we estimate the parameters of biochemical reactions by analyzing available published data.

Our current manuscript is organized into the following sections. Section 1 is the Introduction. In Section 2, the mathematical model is constructed in the form of a deterministic set of ordinary differential equations (ODEs). Its calibration and stochastic implementation via the Gillespie approach are described. Section 3 presents the model-based predictions of the parameter sensitivity of net single-cell HBV production and the characteristics of the variability in viral production, including the secretion of non-infectious viral particles and the sensitivity analysis to drug application. The effects of combining direct-acting antivirals, such as small interfering RNAs, capsid inhibitors and nucleoside analogs, are examined. Overall, the study results are discussed in Section 4 and briefly summarized in Section 5.

## 2. Materials and Methods

### 2.1. Molecular and Genome Structure of HBV

**Figure 1:**
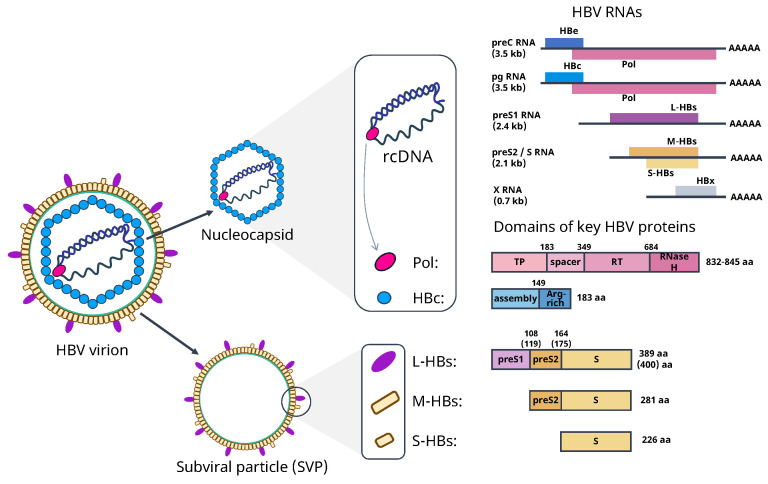
Hepatitis B virion structure and genome organization.

HBV is an enveloped DNA virus with the outer envelope consisting of three types of surface antigens (L-, M- and S-HBs) [2]. The internal viral capsid is composed of either 240 or 180 copies of HBV core (HBc), which is a 23 kDa protein. It is considered to be engaged in many steps of HBV replication. The viral genome is a relaxed circular (rc) partially double-stranded DNA (rcDNA) of about 3200 base pairs. It encodes open reading frames C, P, S and X [11]. The rcDNA is attached to the viral polymerase (Pol), a reverse transcriptase. The organization of HBV is shown in Figure 1.

### 2.2. Intracellular Replication of HBV

The complete HBV virion has an outer envelope layer and a nucleocapsid with rcDNA inside [2,6,19]. HBV infects hepatocytes via sequential interaction with heparan sulfate proteoglycans and the sodium taurocholate co-transporting polypeptide (NTCP) [20]. Following fusion of viral and cellular membranes, the viral particle is internalized (endocytosis) and the viral capsid is released into the cytoplasm and transported via endosomal compartments and cytosol through the nuclear pore to the nucleus. The viral rcDNA, released in the nucleus, is converted into cccDNA, which serves as a template for viral transcription, i.e., generation of pregenomic and subgenomic RNAs, such as pgRNA, precore mRNA, preS1 mRNA, preS2/S mRNA, and X mRNA. The outcome of a single HBV infection cycle includes the secretion of complete virions, empty virions or spheric and filamentous subviral particles containing only the HBsAg [21]. HBx protein is required for HBV replication [11]. It is transported to the nucleus to stimulate cccDNA transcription [21]. The translated core protein (HBc) molecules encapsidate the pgRNA and HBV polymerase, thus forming an icosahedral capsid shell [2]. The packaged pgRNA and Pol is reverse transcribed, giving rise to progeny rcDNA [20]. The newly produced rcDNA genomes may be either enveloped at the endoplasmic reticulum and secreted via multivesicular bodies as HBV or moved back to the nucleus, where they are transformed to cccDNA and maintained at 5 to 12 copies per cell [20,22]. The latter process is called intracellular recycling or amplification. HBV infection is characterized by persistence of cccDNA mini-chromosomes in hepatocytes and partial integration of HBV DNA into the cellular genome. Three types of HBV particles are produced, i.e., (i) infectious virions (42 nm) consisting of surface antigens: large (L-), middle (M-) and small (S-) HBs, surrounding a nucleocapsid, consisting of HBc, Pol and rcDNA; (ii) subviral particles (22 nm) that lack nucleocapsid; and (iii) spheres/filaments consisting of S-HBs proteins [11]. We neglect the impact of the double-stranded linear DNA on the replication process of HBV as the probability of its integration into the host genome is considered to be very low [18,23] and the majority of virions produced by HBV-infected cells package rcDNA-containing nucleocapsids [6,11,21,24]. This could potentially cause the levels of secreted HBsAg predicted by our model to be underestimated [6,21,23].

### 2.3. Deterministic Mathematical Model

**Figure 2:**
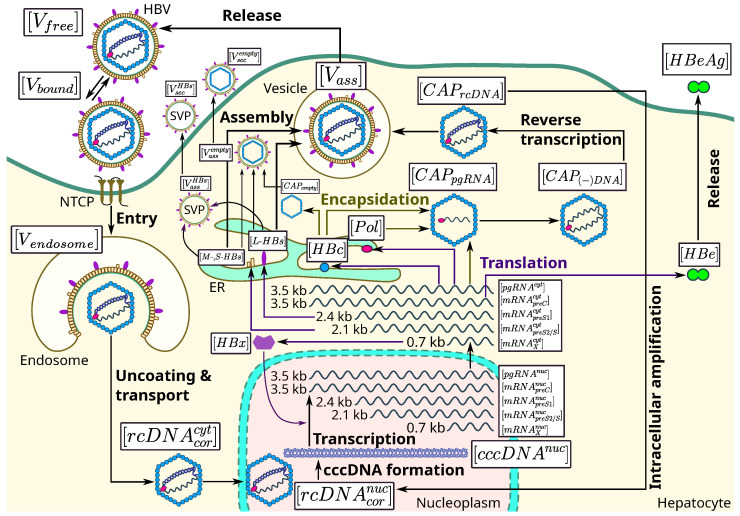
Biochemical scheme of the HBV lifecycle. The individual steps in the HBV lifecycle that are incorporated into the mathematical model are shown schematically. These include attachment, entry, uncoating, trafficking to nucleus, covalently closed circularized DNA (cccDNA) formation, transcription, translation, encapsidation, assembly and secretion [6,19]. Further details are described in the text.

In this section, we formulate both the deterministic and stochastic variants of the mathematical model for the HBV lifecycle. The HBV lifecycle shown in Figure 2 suggests the following set of time-dependent variables listed in Table 2. The ordinary differential equations (ODEs) are used to model the key replication steps, which include [11]:

- HBV entry;
- Covalently closed circular DNA (cccDNA) formation;
- HBV transcription;
- Structural protein translation;
- Encapsidation and DNA synthesis;
- HBV assembly and secretion.

The system of equations is formulated using the basic principles of chemical kinetics, especially the mass action law and Michaelis–Menten parameterization for describing the transcription, replication, coating and assembly processes.

#### 2.3.1. Virus Entry

The virus enters into the human hepatocyte via the NTCP receptor. The rate of change in the number of free, bound and endosome virions is described by the following three ODEs.

(1) $$\frac{d[V_{\mathrm{free}}]}{dt}=-k_{\mathrm{bind}}\left[V_{\mathrm{free}}\right]-d_{V}\left[V_{\mathrm{free}}\right]+k_{\mathrm{diss}}\left[V_{\mathrm{bound}}\right].$$

The first term on the right-hand side describes the binding of free complete virions to the NTCP receptor with the rate constant $k_{\mathrm{bind}}$. The second one corresponds to degradation of the free virion with the constant rate $d_{V}$. The last term depicts the dissociation of bound HBV from sing receptor with the rate constant $k_{\mathrm{diss}}$.

(2) $$\frac{d[V_{\mathrm{bound}}]}{dt}=k_{\mathrm{bind}}\left[V_{\mathrm{free}}\right]-\left(k_{\mathrm{fuse}}+k_{\mathrm{diss}}+d_{V}\right)\left[V_{\mathrm{bound}}\right].$$

The first term describes the increase in cell-bound HBV. The second term considers the fusion of bound virions into endosomes with the constant rate $k_{\mathrm{fuse}}$. The third term depicts the dissociation of bound virions from the NTCP receptor with the constant rate $k_{\mathrm{diss}}$. The last term reflects the degradation of bound virions with the rate constant $d_{V}$.

(3) $$\frac{d[V_{\mathrm{endosome}}]}{dt}=k_{\mathrm{fuse}}\left[V_{\mathrm{bound}}\right]-\left(k_{\mathrm{uncoat}}+d_{\mathrm{endosome}}\right)\left[V_{\mathrm{endosome}}\right].$$

The first term describes the fusion of bound virions with the constant $k_{\mathrm{fuse}}$. The second term represents uncoating of complete virions in endosomes with the constant rate $k_{\mathrm{uncoat}}$, whereas the last term describes the degradation of viruses in endosomes with the constant rate $d_{\mathrm{endosome}}$.

#### 2.3.2. Release of rcDNA and cccDNA Formation

The viral capsid is released into the cytoplasm and transported via endosomal compartments and cytosol through the nuclear pore to the nucleus. The respective equations of the model are listed below (definitions of the parameters follow from the explanation of the model equation terms).

(4) $$\frac{d[{\mathrm{rcDNA}}_{\mathrm{cor}}^{cyt}]}{dt}=k_{\mathrm{uncoat}}\left[V_{\mathrm{endosome}}\right]-\left(k_{\mathrm{trp}}+d_{\mathrm{rcDNA}}\right)\left[{\mathrm{rcDNA}}_{\mathrm{cor}}^{cyt}\right].$$

The first term describes the uncoating of complete virions in endosomes with the constant rate $k_{\mathrm{uncoat}}$; the second term represents the transport of the nucleocapsid along microtubules to the nucleus and the last term describes the degradation of nucleocapsid at the rate $d_{\mathrm{rcDNA}}$.

(5) $$\frac{d[{\mathrm{rcDNA}}_{\mathrm{cor}}^{nuc}]}{dt}=k_{\mathrm{trp}}\left[{\mathrm{rcDNA}}_{\mathrm{cor}}^{cyt}\right]+k_{\mathrm{intrec}}\left[{\mathrm{CAP}}_{\mathrm{rcDNA}}\right]-\left(k_{\mathrm{rep}}+d_{\mathrm{rcDNA}}\right)\left[{\mathrm{rcDNA}}_{\mathrm{cor}}^{nuc}\right].$$

The first term describes the release of capsids with rcDNA into the nucleoplasm, the second term considers the intracellular recycling of rcDNA from the newly formed rcDNA containing capsids, the third term considers the formation of cccDNA organized as a chromatin-like structure [19] and the last term the degradation of rcDNA core particles.

(6) $$\frac{d[{\mathrm{cccDNA}}^{nuc}]}{dt}=k_{\mathrm{rep}}\left[{\mathrm{rcDNA}}_{\mathrm{cor}}^{nuc}\right]-d_{\mathrm{cccDNA}}\left[{\mathrm{cccDNA}}^{nuc}\right].$$

The first term describes the formation of cccDNA by ligation of two DNA strands produced via repair of rcDNA, and the last term reflects the degradation of cccDNA in the nucleus.

#### 2.3.3. Viral RNA Transcription

The transcription of cccDNA in the nucleus generates all viral RNAs required for protein production and viral replication, i.e., the pgRNA, precore RNA, X mRNA, preS1 mRNA and preS2/S mRNA. The respective equations are similar to each other and describe the transcription of the four genes regulated by host factors and the viral X-protein interaction with four open reading frames and also the transport of the mRNA to the cytoplasm and its degradation. HBx protein was shown to be continuously needed to maintain cccDNA in a transcriptionally active state, so we parameterize its effect phenomenologically [25].

(7) $$\begin{array}{cc} \frac{d[{\mathrm{pgRNA}}^{nuc}]}{dt} & =k_{\mathrm{trnsc}}\theta_{\mathrm{HBx}}\ell_{\mathrm{pgRNA}}^{-1}\left[{\mathrm{cccDNA}}^{nuc}\right]-\left(k_{\mathrm{tp}}+d_{\mathrm{pgRNA}}\right)\left[{\mathrm{pgRNA}}^{nuc}\right], \end{array}$$

(8) $$\begin{array}{cc} \frac{d[{\mathrm{mRNA}}_{\mathrm{preC}}^{nuc}]}{dt} & =k_{\mathrm{trnsc}}\theta_{\mathrm{HBx}}\ell_{\mathrm{preC}}^{-1}\left[{\mathrm{cccDNA}}^{nuc}\right]-\left(k_{\mathrm{tp}}+d_{\mathrm{preC}}^{\mathrm{mRNA}}\right)\left[{\mathrm{mRNA}}_{\mathrm{preC}}^{nuc}\right], \end{array}$$

(9) $$\begin{array}{cc} \frac{d[{\mathrm{mRNA}}_{\mathrm{preS}1}^{nuc}]}{dt} & =k_{\mathrm{trnsc}}\theta_{\mathrm{HBx}}\ell_{\mathrm{preS}1}^{-1}\left[{\mathrm{cccDNA}}^{nuc}\right]-\left(k_{\mathrm{tp}}+d_{\mathrm{preS}1}^{\mathrm{mRNA}}\right)\left[{\mathrm{mRNA}}_{\mathrm{preS}1}^{nuc}\right], \end{array}$$

(10) $$\begin{array}{cc} \frac{d[{\mathrm{mRNA}}_{\mathrm{preS}2/S}^{nuc}]}{dt} & =k_{\mathrm{trnsc}}\theta_{\mathrm{HBx}}\ell_{\mathrm{preS}2/S}^{-1}\left[{\mathrm{cccDNA}}^{nuc}\right]-\left(k_{\mathrm{tp}}+d_{\mathrm{preS}2/S}^{\mathrm{mRNA}}\right)\left[{\mathrm{mRNA}}_{\mathrm{preS}2/S}^{nuc}\right], \end{array}$$

(11) $$\begin{array}{cc} \frac{d[{\mathrm{mRNA}}_{X}^{nuc}]}{dt} & =k_{\mathrm{trnsc}}\theta_{\mathrm{HBx}}\ell_{\mathrm{mX}}^{-1}\left[{\mathrm{cccDNA}}^{nuc}\right]-\left(k_{\mathrm{tp}}+d_{{\mathrm{mRNA}}_{X}}\right)\left[{\mathrm{mRNA}}_{X}^{nuc}\right]. \end{array}$$

The following parameterization of the effect of HBx on transcription rates is used:

(12) $$\theta_{\mathrm{HBx}}=\varepsilon_{\mathrm{HBx}}+\left(1-\varepsilon_{\mathrm{HBx}}\right)\frac{\left[\mathrm{HBx}\right]}{\left[\mathrm{HBx}\right]+K_{\mathrm{HBx}}},$$

so that the rate of transcription from cccDNA is constrained between the silenced level $\varepsilon_{\mathrm{HBx}}k_{\mathrm{trnsc}}$ (in the absence of HBx) and the active level $k_{\mathrm{trnsc}}$ (when HBx is present in sufficient amount).

#### 2.3.4. Structural and Envelope Protein Translation

The transcription of viral mRNA in the nucleus is followed by their export to the cytoplasm, where protein translation takes place. The pregenomic RNA is translated to the core protein and the viral polymerase, whereas the envelope proteins and the X-protein are translated from subgenomic RNAs [19]. The corresponding set of equations describing their dynamics are formulated as follows:

(13) $$\begin{array}{cc} \frac{d[{\mathrm{pgRNA}}^{cyt}]}{dt} & =k_{\mathrm{tp}}\left[{\mathrm{pgRNA}}^{nuc}\right]-\left(k_{\mathrm{cap}}F_{\mathrm{cap}}+d_{\mathrm{pgRNA}}\right)\left[{\mathrm{pgRNA}}^{cyt}\right], \end{array}$$

(14) $$\begin{array}{cc} \frac{d[{\mathrm{mRNA}}_{\mathrm{preC}}^{cyt}]}{dt} & =k_{\mathrm{tp}}\left[{\mathrm{mRNA}}_{\mathrm{preC}}^{nuc}\right]-d_{\mathrm{preC}}^{\mathrm{mRNA}}\left[{\mathrm{mRNA}}_{\mathrm{preC}}^{cyt}\right], \end{array}$$

(15) $$\begin{array}{cc} \frac{d[{\mathrm{mRNA}}_{\mathrm{preS}1}^{cyt}]}{dt} & =k_{\mathrm{tp}}\left[{\mathrm{mRNA}}_{\mathrm{preS}1}^{nuc}\right]-d_{\mathrm{preS}1}^{\mathrm{mRNA}}\left[{\mathrm{mRNA}}_{\mathrm{preS}1}^{cyt}\right], \end{array}$$

(16) $$\begin{array}{cc} \frac{d[{\mathrm{mRNA}}_{\mathrm{preS}2/S}^{cyt}]}{dt} & =k_{\mathrm{tp}}\left[{\mathrm{mRNA}}_{\mathrm{preS}2/S}^{nuc}\right]-d_{\mathrm{preS}2/S}^{\mathrm{mRNA}}\left[{\mathrm{mRNA}}_{\mathrm{preS}2/S}^{cyt}\right], \end{array}$$

(17) $$\begin{array}{cc} \frac{d[{\mathrm{mRNA}}_{X}^{cyt}]}{dt} & =k_{\mathrm{tp}}\left[{\mathrm{mRNA}}_{X}^{nuc}\right]-d_{X}^{\mathrm{mRNA}}\left[{\mathrm{mRNA}}_{X}^{cyt}\right]. \end{array}$$

The set of equations for description of the translation of pregenomic RNA, the precore RNA and subgenomic RNAs (for the surface proteins and HBx) are formulated as follows.

(18) $$\begin{array}{c} \frac{d[\mathrm{HBc}]}{dt}=k_{\mathrm{trans}}\ell_{\mathrm{HBc}}^{-1}\left[{\mathrm{pgRNA}}^{cyt}\right]-N_{\mathrm{HBc}}\left(k_{\mathrm{cap}}F_{\mathrm{cap}}\left[{\mathrm{pgRNA}}^{cyt}\right]+k_{\mathrm{cap}}^{\mathrm{empty}}F_{\mathrm{cap}}^{\mathrm{empty}}\right)-\\ -d_{\mathrm{HBc}}\left[\mathrm{HBc}\right], \end{array}$$

(19) $$\frac{d[\mathrm{Pol}]}{dt}=k_{\mathrm{trans}}\ell_{\mathrm{Pol}}^{-1}\left[{\mathrm{pgRNA}}^{cyt}\right]-k_{\mathrm{cap}}F_{\mathrm{cap}}N_{\mathrm{Pol}}\left[{\mathrm{pgRNA}}^{cyt}\right]-d_{\mathrm{Pol}}\left[\mathrm{Pol}\right],$$

(20) $$\begin{array}{c} \frac{d[L\text{-}HBs]}{dt}=k_{\mathrm{trans}}\ell_{\mathrm{HBs}}^{-1}\left[{\mathrm{mRNA}}_{\mathrm{preS}1}^{cyt}\right]-F_{\mathrm{ass}}N_{L\text{-}HBs}(k_{\mathrm{ass}}^{V_{\mathrm{ass}}}\left[{\mathrm{CAP}}_{\mathrm{rcDNA}}\right]+\\ +k_{\mathrm{ass}}^{V_{\mathrm{empty}}}\left[{\mathrm{CAP}}_{\mathrm{empty}}\right])\;-k_{\mathrm{ass}}^{\mathrm{SVP}}F_{\mathrm{ass}}N_{L\text{-}HBs}^{\mathrm{svp}}-d_{L\text{-}\mathrm{HBs}}\left[L\text{-}\mathrm{HBs}\right], \end{array}$$

(21) $$\begin{array}{c} \frac{d[M\text{-},S\text{-}\mathrm{HBs}]}{dt}=k_{\mathrm{trans}}\ell_{S}^{-1}\left[{\mathrm{mRNA}}_{\mathrm{preS}2/S}^{cyt}\right]-F_{\mathrm{ass}}N_{M\text{-},S\text{-}\mathrm{HBs}}(k_{\mathrm{ass}}^{V_{\mathrm{ass}}}\left[{\mathrm{CAP}}_{\mathrm{rcDNA}}\right]+\\ +k_{\mathrm{ass}}^{V_{\mathrm{empty}}}\left[{\mathrm{CAP}}_{\mathrm{empty}}\right])-k_{\mathrm{ass}}^{\mathrm{SVP}}F_{\mathrm{ass}}N_{M\text{-},S\text{-}\mathrm{HBs}}^{\mathrm{svp}}-d_{M\text{-},S\text{-}\mathrm{HBs}}\left[M\text{-},S\text{-}\mathrm{HBs}\right], \end{array}$$

(22) $$\frac{d[\mathrm{HBe}]}{dt}=k_{\mathrm{trans}}\ell_{\mathrm{HBe}}^{-1}\left[{\mathrm{mRNA}}_{\mathrm{preC}}^{cyt}\right]-k_{\sec}^{\mathrm{HBe}}\left[\mathrm{HBe}\right]-d_{\mathrm{HBe}}\left[\mathrm{HBe}\right],$$

(23) $$\frac{d[\mathrm{HBx}]}{dt}=k_{\mathrm{trans}}\ell_{\mathrm{HBx}}^{-1}\left[{\mathrm{mRNA}}_{X}^{cyt}\right]-d_{\mathrm{HBx}}\left[\mathrm{HBx}\right].$$

The following parameterization is used to describe the dependence of the nucleocapsid assembly on the availability of Pol and HBc:

(24) $$F_{\mathrm{cap}}=\frac{\left[\mathrm{Pol}\right]}{\left[\mathrm{Pol}\right]+K_{\mathrm{cap}}N_{\mathrm{Pol}}}\;\cdot \;\frac{\left[\mathrm{HBc}\right]}{\left[\mathrm{HBc}\right]+K_{\mathrm{cap}}N_{\mathrm{HBc}}}.$$

Here, the threshold parameters correspond to the stoichiometry of the nucleocapsid formation [18], $N_{\mathrm{Pol}}=1$, $N_{\mathrm{HBc}}=120$. The dependence of empty capsid assembly on the availability of HBc is described similarly:

(25) $$F_{\mathrm{cap}}^{\mathrm{empty}}=\frac{\left[\mathrm{HBc}Bc\right]}{\left[\mathrm{HBc}\right]+K_{\mathrm{cap}}N_{\mathrm{HBc}}}.$$

To describe the complete virion assembly dependence on the availability of the required surface protein components, the following parameterization is used on the availability of ${\mathrm{CAP}}_{\mathrm{pgRNA}}$, L-HBs and M-,S-HBs.

(26) $$F_{\mathrm{ass}}=\frac{\left[L\text{-}HBs\right]}{\left[L\text{-}\mathrm{HBs}\right]+K_{\mathrm{cap}}N_{L\text{-}\mathrm{HBs}}}\;\cdot \;\frac{\left[M\text{-},S\text{-}\mathrm{HBs}\right]}{\left[M\text{-},S\text{-}\mathrm{HBs}\right]+K_{\mathrm{cap}}N_{M\text{-},S\text{-}\mathrm{HBs}}}$$

where the threshold parameters correspond to the stoichiometry of the complete virion composition [18], $N_{L\text{-}\mathrm{HBs}}=20$, $N_{M\text{-},S\text{-}\mathrm{HBs}}=80$.

The HBs proteins are consumed during virion assembly and the formation of subviral particles (spherical and filamentous) [21]. Based on estimates presented in [18], we assume that a complete virion or a subviral particle consists of about 10 L, 40 S and 10 M protein molecules: $N_{L\text{-}\mathrm{HBs}}^{\mathrm{svp}}=10$ and $N_{M\text{-},S\text{-}\mathrm{HBs}}^{\mathrm{svp}}=50$. Their concentration in blood exceeds that of the complete virions by 2 to 5 orders of magnitude [18,21].

The dimerized HBe protein forms HBeAg particles secreted by the cell. It is considered to be an indicator of active viral replication, which is significantly higher in HBeAg-positive patients [14].

#### 2.3.5. Encapsidation and DNA Synthesis

Availability of viral polymerase and structural proteins is a prerequisite for immature nucleocapsid assembly (pgRNA + Pol + HBc), followed by two-stage reverse transcription synthesizing (−) strand and (+) strand. The pgRNA template is degraded during the (−) strand DNA synthesis, leaving the 5’-terminal RNA fragment, which remains for the subsequent synthesis of (+)DNA and rcDNA [11]. Overall, this results in mature rcDNA containing nucleocapsid. Fractions of them re-enter the nucleus and sustain the formation of cccDNA.

(27) $$\frac{d[{\mathrm{CAP}}_{\mathrm{pgRNA}}]}{dt}=k_{\mathrm{cap}}F_{\mathrm{cap}}\left[{\mathrm{pgRNA}}^{cyt}\right]-\left(k_{(-)\mathrm{RT}}+d_{\mathrm{pgRNA}}^{\mathrm{CAP}}\right)\left[{\mathrm{CAP}}_{\mathrm{pgRNA}}\right].$$

(−)DNA strand formation is described by

(28) $$\frac{d[{\mathrm{CAP}}_{(-)\mathrm{DNA}}]}{dt}=k_{(-)\mathrm{RT}}\left[{\mathrm{CAP}}_{\mathrm{pgRNA}}\right]-\left(k_{(+)\mathrm{RT}}+d_{(-)\mathrm{DNA}}^{\mathrm{CAP}}\right)\left[{\mathrm{CAP}}_{(-)\mathrm{DNA}}\right].$$

The formation of rcDNA-containing nucleocapsids is described by

(29) $$\frac{d[{\mathrm{CAP}}_{\mathrm{rcDNA}}]}{dt}=k_{(+)\mathrm{RT}}\left[{\mathrm{CAP}}_{(-)\mathrm{DNA}}\right]-\left(k_{\mathrm{intrec}}+k_{\mathrm{ass}}^{V_{\mathrm{ass}}}F_{\mathrm{ass}}+d_{\mathrm{rcDNA}}^{\mathrm{CAP}}\right)\left[{\mathrm{CAP}}_{\mathrm{rcDNA}}\right].$$

The formation of empty capsids without pgRNA is described by

(30) $$\frac{d[{\mathrm{CAP}}_{\mathrm{empty}}]}{dt}=k_{\mathrm{cap}}^{\mathrm{empty}}F_{\mathrm{cap}}^{\mathrm{empty}}-\left(k_{\mathrm{ass}}^{V_{\mathrm{empty}}}F_{\mathrm{ass}}+d_{{\mathrm{CAP}}_{\mathrm{empty}}}\right)\left[{\mathrm{CAP}}_{\mathrm{empty}}\right].$$

#### 2.3.6. Virion Assembly and Secretion

Further interaction of rcDNA nucleocapsid with surface proteins results in the formation of complete virions. In parallel, surface proteins form empty subviral particles.

(31) $$\begin{array}{cc} \frac{d[V_{\mathrm{ass}}]}{dt} & =k_{\mathrm{ass}}^{V_{\mathrm{ass}}}F_{\mathrm{ass}}\left[{\mathrm{CAP}}_{\mathrm{rcDNA}}\right]-k_{\sec}^{V_{\mathrm{ass}}}\left[V_{\mathrm{ass}}\right], \end{array}$$

(32) $$\begin{array}{cc} \frac{d[V_{\sec}]}{dt} & =k_{\sec}^{V_{\mathrm{ass}}}\left[V_{\mathrm{ass}}\right]-d_{V}\left[V_{\sec}\right]. \end{array}$$

The following equations describe the formation of subviral particles (HBs spheres/filaments) and empty virions (enveloped empty capsids) in the cytoplasm:

(33) $$\begin{array}{cc} \frac{d[V_{\mathrm{ass}}^{HBs}]}{dt} & =k_{\mathrm{ass}}^{\mathrm{SVP}}F_{\mathrm{ass}}-k_{\sec}^{\mathrm{SVP}}\left[V_{\mathrm{ass}}^{\mathrm{HBs}}\right], \end{array}$$

(34) $$\begin{array}{cc} \frac{d[V_{\mathrm{ass}}^{\mathrm{empty}}]}{dt} & =k_{\mathrm{ass}}^{V_{\mathrm{empty}}}F_{\mathrm{ass}}\left[{\mathrm{CAP}}_{\mathrm{empty}}\right]-k_{\sec}^{V_{\mathrm{empty}}}\left[V_{\mathrm{ass}}^{\mathrm{empty}}\right]. \end{array}$$

Subviral particles, empty virions and HBeAg exit the cell with multivesicular bodies according to the following description:

(35) $$\begin{array}{cc} \frac{d[V_{\sec}^{\mathrm{HBs}}]}{dt} & =k_{\sec}^{\mathrm{SVP}}\left[V_{\mathrm{ass}}^{\mathrm{HBs}}\right]-d_{V^{\mathrm{HBs}}}\left[V_{\sec}^{\mathrm{HBs}}\right], \end{array}$$

(36) $$\begin{array}{cc} \frac{d[V_{\sec}^{\mathrm{empty}}]}{dt} & =k_{\sec}^{V_{\mathrm{empty}}}\left[V_{\mathrm{ass}}^{\mathrm{empty}}\right]-d_{V^{\mathrm{HBs}}}\left[V_{\sec}^{\mathrm{empty}}\right], \end{array}$$

(37) $$\begin{array}{cc} \frac{d[\mathrm{HBeAg}]}{dt} & =k_{\sec}^{HBe}\left[\mathrm{HBe}\right]-d_{\mathrm{HBeAg}}\left[\mathrm{HBeAg}\right]. \end{array}$$

#### 2.3.7. Total Number of Secreted Virions During the HBV Lifecycle

Variable $\left[V_{\sec}\right]\left(t\right)$ indicates the number of mature secreted virions existing outside the cell at time *t*. Note that the total cycle of the modeled infection process takes T=10 days, whereas the half-life of free virions in the intercellular space is about 4 h, i.e., much shorter than the total infection cycle duration. Therefore, the majority of earlier secreted virions have already been degraded: some of them died out and the rest infected other cells. Thus, to characterize the virus net growth and, hence, to evaluate the efficacy of the infection process and its capacity to infect other cells, it is important to calculate the total number of virions released during the infection cycle: $\left[V_{\mathrm{tot}}\right]$.

To this end, we consider the cumulative amount of released virions by time *t*, neglecting their decay outside the cell. We denote this variable as $\left[V_{\mathrm{new}}\right]\left(t\right)$. It is calculated via adding the equation for $\left[V_{\mathrm{new}}\right]$ to the set of the model ODEs:

(38) $$\frac{d[V_{\mathrm{new}}]}{dt}=k_{\sec}^{V_{\mathrm{ass}}}\left[V_{\mathrm{ass}}\right].$$

This equation follows from ODE (31) by neglecting the second term on the right-hand side, responsible for degradation of released virions outside the cell.

The total number of secreted virions is the cumulative amount of virions released during the whole viral replication cycle *T* for a single cell. We take this period to be T≈10 days; therefore,

(39) $$\left[V_{\mathrm{tot}}\right]=\int_{0}^{T}k_{\sec}^{V_{\mathrm{ass}}}\left[V_{\mathrm{ass}}\right]dt=\left[V_{\mathrm{new}}\right]\left(T\right)\approx \left[V_{\mathrm{new}}\right]\left(10\;d\right).$$

### 2.4. Empirical Data for Calibration of the Model

Here, we summarize experimental and clinical data on HBV replication. It provides a quantitative basis for model calibration and needs to be accommodated into the model.

#### 2.4.1. System-Level Infection Dynamics

The acute phase of HBV infection is defined by peak viral load of about $10^{9}$–$10^{10}$ virions per mL of plasma and infection of most hepatocytes [26]. In chronic HBV infection, the viral load is also high, up to $10^{9}$ virions per mL, with infection of 5% to 40% of all hepatocytes [27]. Given that the total number of hepatocytes in human liver is around $2\;\cdot \;10^{11}$ cells [27,28,29,30] and that the liver contains about 12% of the body’s total blood volume under normal conditions [31], the multiplicity of infection (MOI) should not exceed 30–60 virions per cell in vivo.

Plasma concentrations of subviral particles and empty virions are given in [21]: SVPs reach up to 10^14^ copies/mL (5 orders of magnitude larger than viral load, i.e., concentration of complete virions); empty virions reach up to 10^11^ copies/mL (3 orders of magnitude larger than viral load).

HBV-infected hepatocytes live from 10 to 100 days and secrete virions at an average rate of about 1–10 virions per day [27]. The estimated half-lives of infected hepatocytes in HBeAg-positive and HBeAg-negative individuals are 28 and 10 days, respectively [14]. The half-life of HBV virions is approximately 4 h [32].

#### 2.4.2. Data on Virus Entry

The binding kinetics between the NTCP receptor proteins and preS1 (L-HBs) peptides is analyzed in [33]. According to the 1:1 binding model, the following estimates can be made from the binding curves: the dissociation rate is 0.058 ± 0.0037 s^−1^; the association rate is 19000·3500(s · M)^−1^. The virion binding rate is estimated by multiplying the association rate by the preS1 peptide concentration of 100 nM, which is effective for suppressing HBV infection [33]. PreS1 peptide concentrations showing robust interactions with NTCP on binding curves range from 125 to 1000 nM. One HBV virion is considered sufficient to infect a cell because a single rcDNA has been reported to be sufficient to establish productive infection in chimpanzees. However, in most cell culture experiments, this level of efficiency is not reached and a high MOI is used in infection essays [21]. The internalization of virus-like particles takes around 30 min (within half-time of 5 min). Given the typical size of a hepatocyte (25–40 µm) and of its nucleus (6–8 µm), the following transit times can be estimated for a distance between cell membrane and nucleus (9–16 µm): 0.2–0.3 h (pattern (i)), from 0.15–0.5 h (subdiffusion) to 2 h (diffusion) (pattern (ii)), and 13–18 s (pattern (iii)).

#### 2.4.3. Data on cccDNA Formation

The kinetics of HBV trafficking to the nucleus is studied in [34]. The study shows that, after infection with MOI 200, HBV DNA is first detected in the cytoplasm within 1 h, in the nucleus within 3 h, and their levels saturate by 12 h (with 3.5-fold-higher levels in cytoplasm). The HBV DNA is estimated to be integrated in 1 per 1000 cells [35]. The amount of cccDNA at day 6 post-infection is about 0.3–0.45 copies per cell [25]. The following HBV DNA amounts are reported for HepG2 cells transfected with HBV plasmids [36]: 162 ± 10 copies/cell of total rcDNA, of which 25 ± 2 copies/cell (≈15%) are in the nucleus, and 2.6 ± 1.1 copies/cell of cccDNA (≈10% of nuclear rcDNA).

The decay of HBV DNA in nucleus and cytoplasm with the apparent exponential rate around 0.08 h^−1^ begins after the rise in cccDNA (24 h post-infection). The kinetics of the HBV lifecycle in HepG2-NTCP-K7 cells is studied over 42 days post-infection [20]. The estimated cccDNA half-life is approximately 40 days. The estimates for cccDNA half-life have been reviewed in [37]. The amount of cccDNA measured in in vitro infection studies is on average about 2–5 molecules per infected cell [38] and can reach up to 13 copies per infected cell at MOI=1000 [20]. Lower numbers (up to 1–3 copies) are reported in human chronic hepatitis B infection and in infected human liver chimeric mice [3,39].

#### 2.4.4. Intracellular Transport

The patterns of individual intracellular virus-like particle motility are analyzed in [40,41]. Three modes of movement have been distinguished: (i) slow actin-mediated movement with velocities below 0.5 µm/s and active transport (superdiffusion) pattern, (ii) normal and confined motion in cytoplasm with velocities below 1 µm/s and normal/anomalous (diffusion and subdiffusion) patterns, and (iii) directed motion along microtubules with velocities below 4 µm/s and active transport (superdiffusion) pattern.

The intracellular transport of HBV virions and subviral particles (HBsAgs) is shown to be mainly actin-filament-dependent rather than microtubule-dependent [42], while the nucleocapsids are delivered to the nucleus along the microtubules [43]. Virion transport speed on microtubules versus particle size is reviewed in [44]. Given the HBV nucleocapsid size (30–34 nm), the maximum intracellular velocities of nucleocapsids should be around 1–2 µm/s. The study of HBcAg nucleocapsid (virus-like particle) intracellular trafficking shows that nucleocapsids reach the nucleus in about 3 h [45].

The intracellular trafficking of HBsAg particles is studied in [42]. The HBsAg internalization by endocytosis is detected as early as 1–3 min and is mostly finished by 30 min after infection. Projections of intracellular HBsAg particle trajectories onto the plane reveal an anomalous actin-mediated motility pattern, with lower velocities in confinement areas. Most of the estimated velocities of intracellular HBsAg particles are below 0.1 µm/s, although a small number of trajectories (up to 20%) exhibited speeds up to 0.6 µm/s.

#### 2.4.5. Data on RNA Transcription

As presented in [11], cccDNA produces pregenomic RNA (3.5 kb), translated into HBc (183 aa) and Pol (845 aa) proteins and four different HBV RNAs: (i) precore mRNA (3.5 kb), translated into HBe (159 aa); (ii) preS1 mRNA (2.4 kb), translated into L-HBs (400 aa); (iii) preS2/S mRNA (2.1 kb), translated into M-HBs (281 aa) and S-HBs (226 aa); and (iv) X mRNA (0.7 kb), translated into HBx (154 aa). We have estimated transcription and translation rates based on the lengths of the cccDNA or pgRNA/mRNA genome encoding the corresponding transcript or protein and the baseline elongation rates in eukaryotic cells: 25 nucleotides per second for transcription [46] and 200 aa/min for translation [18,47]. The degradation rates of 3.5 kb RNA (pgRNA and preC mRNA), 2.1–2.4 kb RNA (preS1/preS2/S mRNA) and 0.7 kb (X mRNA) have been estimated using Northern blot data presented in [42,48,49].

The role of HBx on HBV replication is reviewed in [50]. HBx is needed for initiating and maintaining transcription from cccDNA. Infection of primary human hepatocytes with HBx-deficient viral particles resulted in undetectable levels of HBV pgRNA, rcDNA, virus progeny, HBeAg and HBsAg [25]. Infection of HepaRG cells with HBx-negative viruses results in a significant reduction in pgRNA levels in comparison with infection with wild-type (WT) HBV (about 2.5-fold reduction 5 days post-infection and 6.5-fold reduction 10 days post-infection) and barely detectable rcDNA, virus progeny, HBeAg and HBsAg. Other studies show similar effects in HepG2 cells using transfection techniques with HBx-negative or HBx mutant expression plasmids [51,52]. Although HBV progeny are still released in these experiments, transfection of HepG2 cells with HBx-deficient plasmids results in reduced levels of HBV RNA, encapsidated rcDNA and HBsAg at 48 h post-infection in comparison with transfection with WT HBV plasmids: about 5.5-fold reduction in total HBV RNA, 2–2.5-fold reduction in encapsidated rcDNA and 3.5-fold reduction in HBsAg. The induction of HBx expression in HepaRG cells [25] or introduction of small amounts of HBx into the nucleus of HepG2 cells [51] results in restoration of viral replication. With the chosen parameterization of function $\theta_{\mathrm{HBx}}$, we have calibrated the transcription rate $\varepsilon_{\mathrm{HBx}}k_{\mathrm{trnsc}}$ in HBx-deficient infection to reach the comparable fold reductions. The threshold amount of HBx required for active transcription from cccDNA, $K_{\mathrm{HBx}}$, is set equal to the level of HBx in the cytoplasm observed within 24 h post-infection.

#### 2.4.6. Data on Viral Protein Translation and DNA Synthesis

The rate of nuclear export of RNA molecules is chosen following the estimate from [53]. Previous modeling studies [17,18] considered a negative feedback mechanism via the L-HBs protein: low availability of L-HBs increases reimport of newly produced rcDNA into the nucleus, while high availability facilitates rcDNA export. Although this effect is crucial for duck HBV replication, it is much less prominent for HBV [36,54], so we do not include it in our model. The value for the cytoplasmic HBV RNA increase is chosen to counteract a slight degradation-associated decline in cccDNA level at the end of the lifecycle. The translation rate is taken to be 200 aa/min [18,24,47].

The rates of formation of (−) and (+) DNA strands during reverse transcription are chosen similarly to the reverse transcription rate estimated in [18], assuming that the first interval, at which the (−) strand is synthesized (along with the removal of pgRNA), is twice longer than the second interval at which the (+) strand is synthesized.

#### 2.4.7. Data on Assembly and Secretion of Viral and Subviral Particles

The stoichiometry of virion formation is characterized by the following ratio: rcNC+80S+20M+20L [18], where NC stands for nucleocapsid. The assembly and release stages are calibrated to reach the estimated target levels of secreted particles and molecules, taking into account that the assembly rate of complete virions should be coordinated with the rate of the two-stage reverse transcription process because the amount of capsids containing pgRNA or (−)DNA is negligibly small.

**Figure 3:**
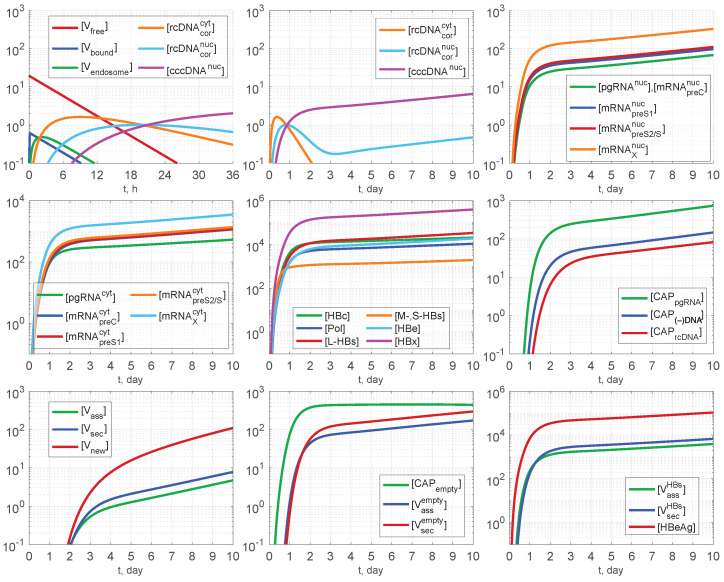
Reference model solution with parameters estimated in Table 3, $\mathrm{MOI}\equiv \left[V_{\mathrm{free}}\right]\left(0\right)=20$.

Overall, the parameters of the model are quantified to match the model solution to empirical data, with initial guesses for the model parameters based on previous studies of virus growth in cells [55], the HBV lifecycle as presented in [12,13,14,15,17,18], and SARS-CoV-2 and HIV-1 replication [53,56]. The overall set of parameters is presented in Table 3. The corresponding solution of the deterministic model predicting the replication dynamics of HBV in a single replication cycle is shown in Figure 3.

### 2.5. Sensitivity Analysis

Sensitivity analysis is used to predict the dependence of HBV production by an infected cell on variations in the rates of underlying biochemical processes. To this end, we consider the total number of new virions secreted by an infected cell during lifecycle time *T* (10 days) from the beginning of the infection (thus disregarding their degradation), i.e., the parameter [$V_{\mathrm{tot}}$] introduced in Section 2.3.7 by Equation (39). This value is a function of the initial condition $\mathrm{MOI}\equiv \left[V_{\mathrm{free}}\right]\left(0\right)$ and parameters of the model listed in Table 3, which can be organized into a vector p:

(40) $$p=\left[p_{1},p_{2},p_{3}\ldots ,p_{K}\right]=\left[k_{\mathrm{bind}},d_{V},k_{\mathrm{diss}}\ldots ,d_{\mathrm{HBeAg}}\right].$$

To calculate sensitivity to parameter $p_{k}$, we employ the following dimensionless measure, called the parameter sensitivity index:

(41) $$S_{k}=\frac{\partial [V_{\mathrm{tot}}]}{\partial p_{k}}\frac{p_{k}}{\left[V_{\mathrm{tot}}\right]},\;k=1,\ldots ,K,\;K=59.$$

This index shows approximately by what percentage the total number of secreted virions will change if the $p_{k}$ parameter changes by 1% relative to its baseline value. The indices form the sensitivity vector $S=[S_{1},S_{2},S_{3},\ldots ,S_{K}]$.

### 2.6. Stochastic Modeling Algorithm

The deterministic model of HBV replication described by the set of ODEs (1)–(38) does not account for the discreteness of the biological system: the component populations must take only integer values. It also does not account for the stochastic nature of the infection process. The most natural way to account for both these factors is to translate the deterministic model into a stochastic Markov chain-based description using the approach proposed and developed by Gillespie [59,60]. With this approach, there is no need for additional parameters other than the coefficients in ODEs (1)–(38).

A Markov chain represents a set of elementary transitions with the corresponding propensity for every transition. Propensity $a_{m}$ for the *m*th transition means that the probability for this transition to occur within an infinitesimal time interval dt equals $a_{m}dt$.

For example, from the first terms on the right-hand side of Equations (1) and (2),

$$\frac{d[V_{\mathrm{free}}]}{dt}=-k_{\mathrm{bind}}\left[V_{\mathrm{free}}\right]-\cdots ,\;\frac{d[V_{\mathrm{bound}}]}{dt}=k_{\mathrm{bind}}\left[V_{\mathrm{free}}\right]-\cdots$$

we can obtain the 1^st^ transition $\left[V_{\mathrm{free}}\right]\to \left[V_{\mathrm{free}}\right]-1,\left[V_{\mathrm{bound}}\right]\to \left[V_{\mathrm{bound}}\right]+1$ with the propensity $a_{1}=k_{\mathrm{bind}}\left[V_{\mathrm{free}}\right]$. The same approach can be applied to all other transitions.

The Gillespie-based Markov chain (MC) of the HBV replication, derived using the elementary reaction terms in deterministic Equations (1)–(38), is shown in Table 4, where all 73 transitions and the corresponding propensities are listed. A formal mathematical notation is used for the time-dependent variables, which is more suitable for the description and implementation of the stochastic Markov chain Monte Carlo (MCMC) model. The parameters and functional forms of the calibrated reaction kinetics are transformed into the propensity functions of the respective elementary reactions following the Gillespie approach.

Variables of the stochastic model can have only non-negative integer values. The following notations for transitions are used in Table 4 for brevity:

$$\begin{array}{cc} \left[V_{\mathrm{free}}\right]\to \left[V_{\mathrm{free}}\right]-1 & \;\Leftrightarrow \;{\left[V_{\mathrm{free}}\right]}^{--}\\ {}[V_{\mathrm{bound}}]\to \left[V_{\mathrm{bound}}\right]+1 & \;\Leftrightarrow \;{\left[V_{\mathrm{bound}}\right]}^{++}; \end{array}$$

i.e., superscripts “^−−^” and “^++^” denote, respectively, the decrease and increase in abundance of the corresponding component by one. The same applies to all other transitions in Table 4.

To implement the dynamic MC description numerically, a number of methods (stochastic simulation algorithms) are available, including the popular Gillespie’s direct method [59,60], which we employ here, and a number of exact and approximate SSA variations [61]. In Gillespie’s direct method, for every time step, we have to generate two random numbers, $r_{1}$ and $r_{2}$, uniformly distributed in the interval [0,1]. The first number determines the time interval for this step: $\Delta t=-\ln(r_{1})/A$, where $A=\sum_{m=1}^{M}a_{m}$ is the sum of all propensities and $a_{m}$ is the propensity of the *m*-th transition. The second number determines the transition to be performed at this step. To this end, a cumulative sum of all propensities is calculated: $A_{m}=\sum_{k=1}^{m}a_{k}$; then, the smallest index *m* satisfying the non-equality $A_{m}>r_{2}A$ has to be found, which specifies the index *m* of transition to be performed. After this, the abundances of corresponding components have to be updated as well as the propensities, and the current time *t* should be increased by Δt. The process continues until the final time $t_{\mathrm{final}}$ is reached.

To obtain statistically reliable results, a large number of simulations are needed (typically $10^{5}$–$10^{6}$ realizations). Therefore, acceleration of the computation is crucial for this analysis. Previously, we have proposed the hybrid stochastic–deterministic method [62,63,64,65,66] to accelerate computations. In this method, the stochastic dynamics of any component can be switched automatically to the deterministic dynamics as soon as its abundance exceeds a certain predetermined threshold. The reason is as follows: when the abundances are large, the propensities and their sum *A* are large too; therefore, the time-stepping interval Δt becomes extremely small, and the computation time increases essentially. At the same time, the stochastic dynamics of a component with large abundance can be rather accurately approximated by a deterministic model described by the corresponding equation from the ODE set (1)–(38). The computations show that, when the threshold is set to be $2\times 10^{4}$, then the computation time is decreased by a factor of five, whereas the main statistical characteristics, such as mean value, median and quantiles, have negligible discrepancy from the results obtained by the fully stochastic numerical scheme.

## 3. Results

### 3.1. Sensitivity Analysis of the Deterministic Model

To calculate the sensitivity indices, the parameters of ODEs (1)–(38) are varied one-by-one by 0.1% relative to their baseline values in both directions. Then, the ODEs (1)–(38) are integrated numerically, and the derivative in (41) is evaluated by central differences.

The results of the numerical computation of the sensitivity vector $S=[S_{1},S_{2},\ldots ,S_{K}]$, i.e., a vector of sensitivity indices defined by Equation (41) in Section 2.5, are presented in Table 5. The parameters in the table are ranked by the absolute value of $|S_{k}|$.

Negative values of $S_{k}$ mean that the total number of secreted virions decreases with an increase in parameter $p_{k}$. The listed values indicate requirements for accuracy of determination of various parameters in the mathematical model. The most influential processes are represented by the coefficients

- $d_{pgRNA}^{\mathrm{CAP}}$—degradation rate of pgRNA-containing capsids;
- $k_{\mathrm{trnsc}}$—active transcription rate from cccDNA;
- $l_{\mathrm{pgRNA}}$—length of HBV genome coding pgRNA;
- $k_{\mathrm{fuse}}$—fusion and entry rate of endocytosis;
- $d_{V}$—degradation rate of free extracellular virions;
- $k_{\mathrm{bind}}$—binding rate of virion to NTCP receptor;
- $k_{\mathrm{diss}}$—dissociate rate of bound virions;
- $k_{\mathrm{trp}}$—transport of rcDNA to nucleus;
- $d_{\mathrm{pgRNA}}$—degradation of pgRNA in cytoplasm;
- $k_{(-)\mathrm{RT}}$—synthesis rate of (−) strand DNA;
- $k_{(+)\mathrm{RT}}$—synthesis rate of (+) strand DNA.

The magnitude of the sensitivity parameter $S_{k}$ for all these coefficients exceeds one. It follows that entry, transcription, transport and DNA/RNA degradation processes have much stronger effects on virus production than the other coefficients. Hence, they can be considered as the best for designing antiviral drugs.

### 3.2. Variability of the HBV Lifecycle Due to Stochasticity

**Figure 4:**
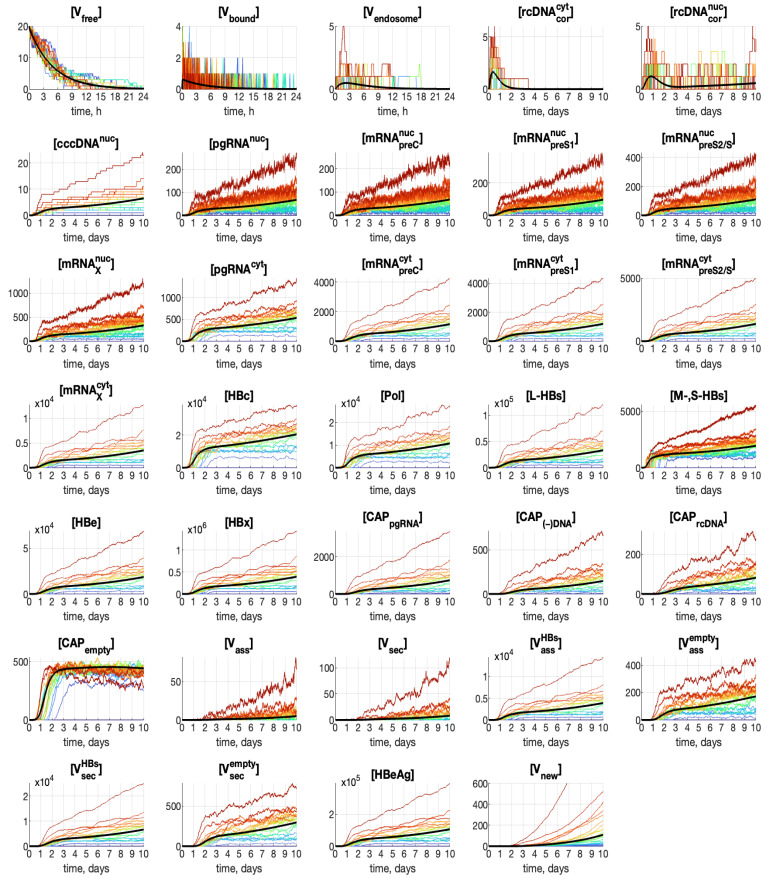
Examples of 20 stochastic trajectories for all model components and total number of produced viruses [V_cum_]. Initial condition is $\left[V_{\mathrm{free}}\right]\left(0\right)\equiv \mathrm{MOI}=20$. The higher is $\left[V_{\mathrm{tot}}\right]=\left[V_{\mathrm{cum}}\right]\left(10\;d\right)$ — the closer is the color of the trajectory to the red end of spectrum. The black curves indicate the deterministic trajectories.

An example of 20 stochastic realizations of the stochastic HBV model is presented in Figure 4. The deterministic trajectories are also shown in the figure; they are indicated by bold black curves.

The color of the stochastic trajectories is selected such that the trajectories with the greater [$V_{\mathrm{tot}}$], defined by Equation (39), have the color closer to the red end of the color spectrum. One can observe that realizations with a high final output, i.e., large [$V_{\mathrm{tot}}$], are characterized by higher amplitudes already at the early stages of the replication process: the effect can be seen already for [${\mathrm{cccDNA}}^{nuc}$].

The dynamics of [${\mathrm{pgRNA}}^{nuc}$], [${\mathrm{mRNA}}_{\mathrm{preC}}^{nuc}$], [${\mathrm{mRNA}}_{\mathrm{preS}1}^{nuc}$], [${\mathrm{mRNA}}_{\mathrm{preS}2/S}^{nuc}$] and [${\mathrm{mRNA}}_{\mathrm{preX}}^{nuc}$] demonstrate rapid high-amplitude fluctuations.

Trajectories for some components, having high abundance, have the tendency to form clusters around certain values. This can be noticed for components from [${\mathrm{mRNA}}_{X}^{nuc}$] to [L-HBs] and also for [HBe] and [HBx]. Clustering also takes place for fast-fluctuating components, mentioned in the previous paragraph, but the clusters are not visible against the background of the fluctuations. The trajectories for [${\mathrm{cccDNA}}^{nuc}$] show a quasi-steady-state pattern. However, this feature is not identical to clustering: the trajectories follow integer values because of the discrete nature of the reacting biochemical species. Nevertheless, discreteness at low numbers of [${\mathrm{cccDNA}}^{nuc}$] determines the locations of trajectory clusters for other highly populated components, which start developing after the emergence of [${\mathrm{cccDNA}}^{nuc}$]. Thus, clustering is associated with the formation of varying numbers of [${\mathrm{cccDNA}}^{nuc}$] molecules.

**Figure 5:**
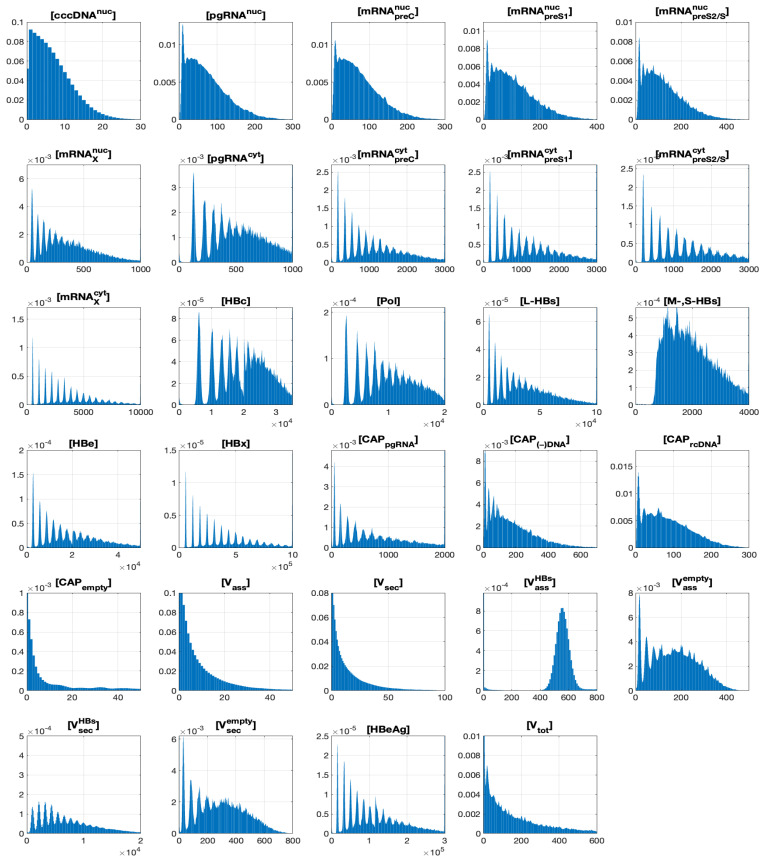
Histograms for non-decaying components at t=10 days and for HBV entering the cell (MOI=20). The histograms are normalized to approximate the probability density functions (PDFs).

Normalized histograms for all the components, which persist until the end of the simulation time, are presented in Figure 5. They are calculated for time t=10 days and MOI=20. Every histogram is normalized by the product of the interval width and the number of samples so that the total area of each histogram equals unity. Therefore, every histogram approximates the probability density function (PDF) for the distribution of the corresponding component.

One can see that the histogram shapes are far from Gaussian. Only the histogram for [$V_{\mathrm{ass}}^{\mathrm{HBs}}$] is bell-shaped, which resembles the normal or log-normal distribution. The histograms for [V_ass_], [V_sec_] and [V_tot_] look to be close to exponential curves, although the histogram for [V_tot_] has a noticeable peak for small values of argument. Most components have multiple peaks. These peaks correspond to the discrete numbers of synthesized [cccDNA^*nuc*^] molecules. The number of peaks is related to the initial number of infecting virions, i.e., MOI.

**Figure 6:**
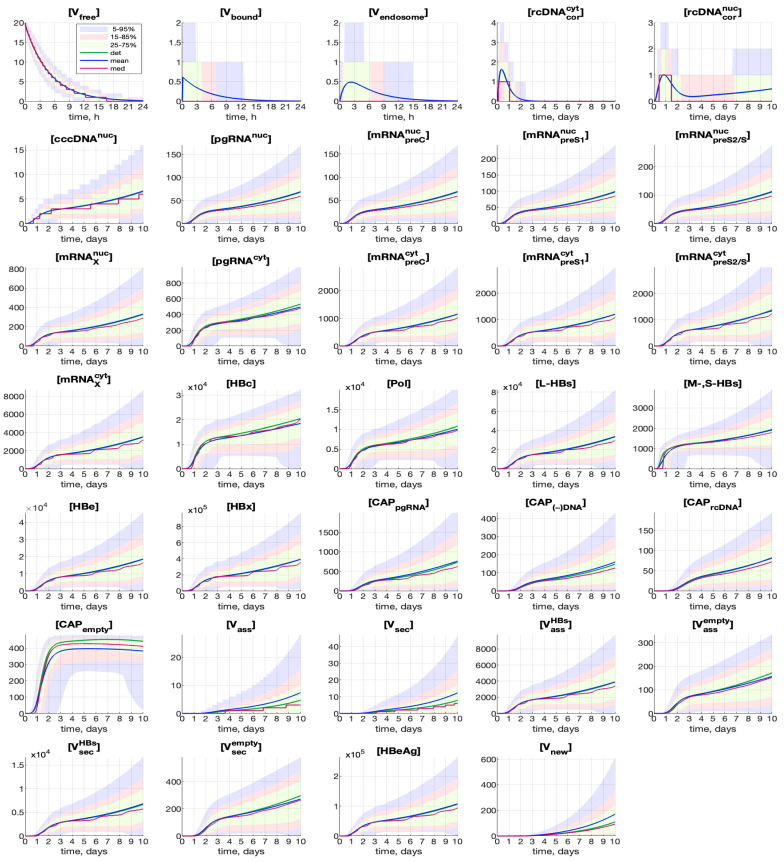
Time-varying confidence intervals for all components for MOI=20. The deterministic solutions (det), mean values (mean) and medians (med) of the stochastic ensemble realizations are plotted. The 25–75% confidence intervals (which include 50% of all realizations) are indicated by light-green patches. The 15–85% confidence intervals are shown by the pink patches. The 5–95% confidence intervals (which include 90% of all realizations) are shown by the light-blue patches.

As the histograms are far from Gaussian, to characterize the sample statistics, it is more appropriate to compute the mean and median values and the confidence intervals. The time variations of the mean and median for every component are shown in Figure 6 by blue and red curves, respectively, computed for the initial condition MOI=20. Here, for comparison, the deterministic solutions are shown as well by green lines. For most components, the mean value is close to the deterministic solution. Nevertheless, a much greater discrepancy can be noticed for the components determining the productivity of the infection process: [V_ass_], [V_sec_] and [V_tot_].

The confidence intervals are presented in Figure 6 in the form of colored patches. The 25–75% confidence intervals (which include 50% of all the realizations) are indicated by light-green patches. The 15–85% confidence intervals are shown by pink patches. They overlap with the 25–75% confidence intervals. The widest 5–95% confidence intervals (which include 90% of all the realizations) are shown by light-blue patches. Note that the confidence intervals turn out to be quite wide, as do the histograms in Figure 5.

**Figure 7:**
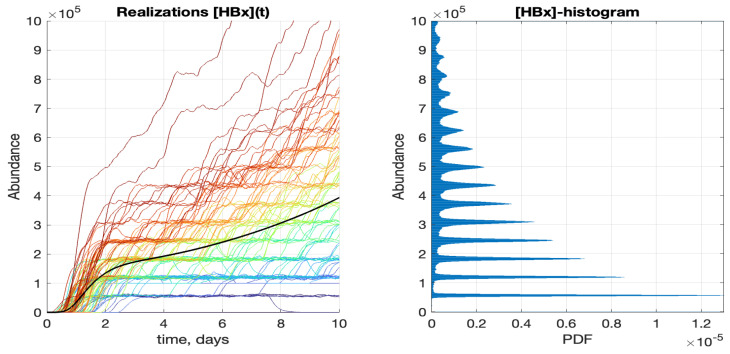
(**Left**): An example of 100 stochastic trajectories for [HBx]. The line colors are ordered along the color spectrum in accordance with [V_tot_] value for a given trajectory: the red end corresponds to the highest [V_tot_]. (**Right**): Normalized histogram for [HBx] at the end (t=10 days) of the infection cycle with swapped axes.

The correlations between the positions of peaks and trajectory clusters are shown in Figure 7, where the [HBx] component is considered as an example. The graphs are drawn at a larger scale than in Figure 4 and Figure 5, so the patterns are more visible. Note that the peak positions of the histogram are at the same abundance values as the clustering locations of the trajectories. As mentioned above, clustering is caused by the discreteness of low-populated components produced at the early stages of the infection cycle.

### 3.3. Infection Productivity and Efficiency

As mentioned above, the total number of secreted mature virions defined by Equation (39) can serve as a measure of the single cell infection productivity. This is because knowing this value is necessary (although it is not sufficient) to calculate how many healthy cells a given infected cell can contaminate. To calculate the total number of free mature virions released by a large number of cells, we need to multiply the total number of infected cells by the average of the total number of virions produced by each cell. Thus, in the stochastic case, the mean value [V_tot_] appears to be a reasonable characteristic of the single cell infection productivity.

**Figure 8:**
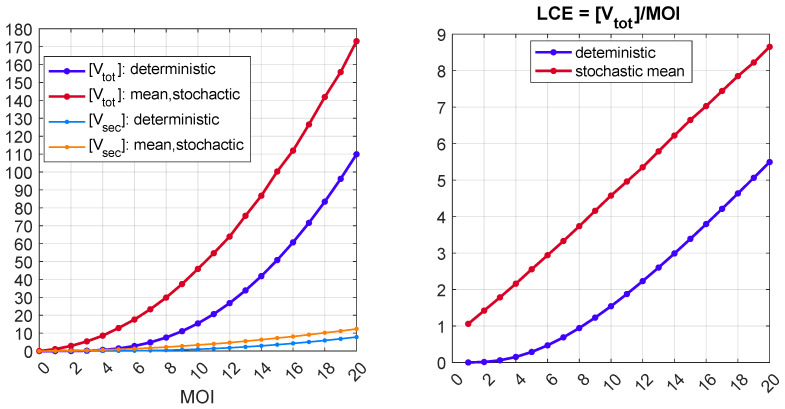
(**Left**): Dependence of [V_tot_] and [V_sec_] on MOI in the framework of the deterministic model and their mean values in the stochastic case, as explained in the legend. (**Right**): Lifecycle efficiency of the infection process in the deterministic (blue) and stochastic (red) cases.

The dependence of [V_tot_] on MOI, predicted by the deterministic model, and its mean value, computed by the stochastic version of the model, are shown in Figure 8 (left). Here, the value of [V_sec_] and its stochastic mean are also plotted. One can see that the stochastic mean [V_tot_] essentially exceeds its deterministic value: 173 against 110 with MOI=20. This difference suggests a critical role of the discreteness and stochasticity of the biochemical processes in the lifecycle of HBV. The efficiency of the HIV-1 lifecycle can be characterized by the ratio of the total viral progeny to the number of virions infecting the cell (MOI). This characteristic is defined as the lifecycle efficiency (LCE) in our previous studies [65,66]:

(42) $$\mathrm{Life}\;\mathrm{Cycle}\;\mathrm{Efficiency}\;\equiv \mathrm{LCE}=\frac{\mathrm{mean}(\left[V_{\mathrm{tot}}\right])}{\mathrm{MOI}}.$$

The dependence of LCE on MOI, computed by the deterministic and stochastic models, is shown in Figure 8 (right). One can observe a significant quantitative difference between the predictions of the two models. In addition, the pattern of dependence is different, i.e., a quadratic law of LCE when MOI→1 predicted by the deterministic model versus a linear dependence of LCE on MOI in the framework of the stochastic model. For example, at MOI=1, the deterministic model gives a value of LCE = 0.0006, i.e., practically zero, while the stochastic model estimates it as 1.06, i.e., greater than one. Note that the backward continuation of this straight red line in Figure 8 (right) does not pass through the origin but goes slightly above it.

### 3.4. Probability of Productive HBV Infection

**Figure 9:**
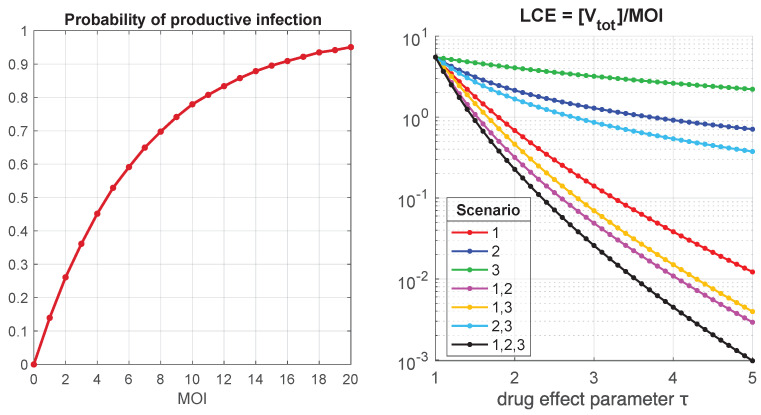
(**Left**): Probability of a productive HBV infection as a function of MOI. (**Right**): Effects of antivirals (siRNAs, nucleoside analogues, and capsid inhibitors) and their combinations on lifecycle efficiency of the infection process for various scenarios of drug combinations (explained in the legend).

The deterministic model described by the set of ODEs (1)–(38) always generates a single nonzero output for the number of produced virions, i.e., $[V_{\mathrm{tot}}]>0$ for any finite initial condition MOI>0. According to the stochastic model, no virions can be released by an infected cell in some realizations even for finite values of MOI. Such realizations represent degenerate or extinct infection cycles. The stochastic model described by MC in Table 4 enables computing the probability of extinct cases, $P_{e}$, and the corresponding probability of a developed infection process, $P=1-P_{e}$. The computed dependence of the probability of productive infection, *P*, on the initial number of infecting virions (MOI) is presented in Figure 9 (left). One can see that, for MOI<5, the single-cell HBV infection will be productive and lead to the generation of new infectious virions in less than 50% of cases.

### 3.5. Sensitivity to Drug Combinations

To analyze the sensitivity of virion production by an infected hepatocyte to multi-component antiviral effects, we evaluate the change in the total number of secreted viruses for various combinations of the following antiviral drugs: siRNAs, nucleoside analogues and capsid inhibitors [6]. Phenomenologically, their impact is on the processes characterized in the model equations by a set of coefficients, p, in the ODEs (1)–(38), listed below. To this end, we study the dependence of the total number of secreted virions on a simultaneous variation of the parameters following the idea of a directional derivative (Gâteaux derivative [67]).

Let $\left[V_{\mathrm{tot}}\right]\left(p*\right)$ be the total number of secreted virions for the baseline set of parameters. We form a vector of components from those model parameters that reflect the drug effects. We introduce parameter τ, which is associated with the drug concentration and its effect on the respective model parameter(s); i.e., it parameterizes the degree of variation of corresponding coefficients.

Three different antiviral drugs are considered. We divide the parameters to be varied into three groups accordingly:

**Group** **1:** Small interfering RNAs destroy viral RNAs; they affect five parameters, $d_{\mathrm{pgRNA}}$, $d_{\mathrm{preC}}^{\mathrm{mRNA}}$, $d_{\mathrm{preS}1}^{\mathrm{mRNA}}$, $d_{\mathrm{preS}2/S}^{\mathrm{mRNA}}$, and $d_{X}^{\mathrm{mRNA}}$, in Equations (7)–(11) and (13)–(17); they increase with the application of the drug.
**Group** **2:** Nucleoside analogues block the synthesis of (−) DNA strands; this is related to the $k_{(-)\mathrm{RT}}$ parameter in Equations (27) and (28); it decreases under drug application.
**Group** **3:** Capsid inhibitors block capsid formation; they affect the parameter $k_{\mathrm{cap}}$ and the parameter $k_{\mathrm{cap}}^{\mathrm{empty}}$ in Equations (13), (18), (19), (27) and (30), which decrease after drug application.

These drugs are classified in Phase 2 clinical trials (Groups 1 and 3) or approved for use (Group 2). Next, we examine the efficacy of the antivirals added in mono-, duo- and triple combinations for the following seven scenarios:

**Scenario** **1:** Only coefficients from Group 1 change. They increase simultaneously by a factor of τ.
**Scenario** **2:** Only coefficient from Group 2 changes. It decreases proportionally to 1/τ.
**Scenario** **3:** Only coefficients from Group 3 change. They decrease simultaneously in proportion to 1/τ.
**Scenario** **1,2:** Coefficients from Groups 1 and 2 change (in opposite directions).
**Scenario** **1,3:** Coefficients from Groups 1 and 3 change (in opposite directions).
**Scenario** **2,3:** Coefficients from Groups 2 and 3 change. They decreases simultaneously in proportion to 1/τ.
**Scenario** **1,2,3:** Coefficients from all Groups 1, 2 and 3 change simultaneously in proper directions.

The results of the computations are presented in Figure 9 (right), where one can find seven curves: LCE versus τ for all seven above scenarios corresponding to mono- and combined use of the antivirals. It appears that the application of small interfering RNA molecules has a very strong effect, whereas the capsid inhibitor shows a modest impact. A combination of multiple drugs leads to a multiplicative rather than additive effect on the reduction in virus production.

## 4. Discussion

In this work, a mathematical model of HBV replication in infected hepatocytes is developed. It is formulated following deterministic and stochastic frameworks to examine the sensitivity of biochemical stages to parameter variations and the probability of productive versus degenerate infections. The model considers the dynamics of 33 time-dependent variables ranging from entry of virions into the cell to secretion of viral particles. Further extension of the model will be the focus of our future work. The lifecycle of HBV should be considered in the context of host factors that restrict viral replication, including interferon alpha [68] and hepatic metabolism [69]. Note that degradation and death (apoptosis) of the cell due to virus production are not yet included in our and similar models [17,18]; therefore, the modeling is restricted by the typical lifecycle. Modeling of cell apoptosis requires serious further study, which will be carried out in the future.

The parameter values presented in Table 3 are derived using three types of sources: (i) experimental data as detailed in Section 2.4, (ii) estimates suggested by other mathematical models and (iii) individual tuning to ascertain the observed scale of the virus production (the target numbers presented in Section 2.4). Table 5 shows the results of the sensitivity analyses for MOI = 20. This value is consistent with a scale of the infectious virions produced by a single infected hepatocyte in ten days, as discussed in Section 2.4. The effect of lower MOI is further explored in Figure 8 and Figure 9.

The empirical data used for the calibration of the model are detailed in Section 2.4. The estimates of the model parameters are based on (i) previous modeling works and (ii) direct estimates of the parameters from the experimental data (e.g., binding and dissociation rates of extracellular virions, lengths of the HBV genome encoding various transcripts and proteins, transcription and translation rates, and degradation rates for transcripts, proteins, and intracellular transit rates) and (iii) indirect estimates obtained during model calibration to match the kinetics and levels of model variables to the generalized picture of the HBV lifecycle based on the information available in the cited literature. The references include various reviews, as well as experimental peer-reviewed works that use different infection and transfection protocols on multiple cell lines and HBV plasmids (including specific protein-deficient or mutant expression plasmids to calibrate the protein regulation aspects of HBV replication). The detailed descriptions of various aspects are included in Section 2.4 and Table 3.

The formulated mathematical model of the HBV lifecycle consists of differential equations characterizing the time course of the elementary biochemical stages of virus generation. The proposed resolution reflects the aim to apply the model for predicting the targets of antiviral therapy. The sensitivity analyses of the individual stages regarding net virus production help to identify the molecular targets for drug development. The equations represent a chain of sequential reactions that are non-linearly (cross-)regulated, as visualized in Figure 2. Overall, the set of calibrated equations is a consistent model that can be reduced for a specific purpose by potential users afterwards.

The model represents an in silico description of the HBV–hepatocyte interaction in a single cell. It can be used to examine the pharmacodynamics of drugs from various clinical phases: preclinical, Phase I, Phase II and approved [6]. It has been used to rank the antiviral drug efficacy according to the reductions in the value of the lifecycle efficacy of HBV infection. The model predicts that the individual antiviral effects of the three considered drugs, i.e., siRNAs, nucleoside analogues and capsid inhibitors, synergize multiplicatively. The siRNA molecules have a very strong reduction effect on viral production, which exceeds, by about two orders of magnitude, the effects of the two other antivirals. Other drugs could be assessed in a similar fashion.

Therefore, biochemical reactions strongly affecting the virus production and synergy effects of two to three antiviral drug combinations have been investigated in our work. The dose-dependent effects of drug combinations on the probability of productive infection suggest that a complete reduction in HBV generation by infected cells is feasible. However, the extrapolation of the results to chronic HBV infection requires a multiscale extension of the model, i.e., the description of hepatocytes and immune cell dynamics.

Today, the functional cure of chronic HBV infection is considered to be equivalent to an acute resolved infection [6]. Combination therapies including direct-acting antivirals, immunomodulators and therapeutic vaccines are considered to be methods of curing chronic HBV infections [4,21]. However, some concerns exist regarding the performance of such therapies tested so far [8,9]. In our view, mathematical modeling provides relevant tools to address a range of issues related to combination therapies, including the pleiotropic effects of drugs, their toxicity and non-linear interactions. To this end, multiscale mathematical models are to be developed and applied to analyze the results of clinical studies. The HBV lifecycle model presented in our study provides a calibrated building module for such multidisciplinary approaches.

Thus, the developed model represents a theoretical tool for predicting the response of infected hepatocytes to antiviral drugs and designing optimal regimes of drug combination to assist clinicians in treatment navigation for functional curing chronic hepatitis B infection [10].

## 5. Conclusions

A detailed calibrated mathematical model of the HBV lifecycle is developed. It predicts the sensitivity of net virus production by infected cells to variations in the replication stages. The efficacy of approved and Phase II antivirals on HBV replication is quantified, demonstrating a very strong effect of siRNA molecules compared to capsid inhibitors and nucleoside analogues.

The empirical data have been used for the calibration of the model, as detailed in Section 2.4. The estimates of the model parameters are based on (i) previous modeling works, as well as on (ii) direct estimates of parameters from experimental data (e.g., binding and dissociation rates of extracellular virions, lengths of the HBV genome encoding various transcripts and proteins, transcription and translation rates, degradation rates for transcripts and proteins, and intracellular transit rates) and (iii) indirect estimates obtained during model calibration to match the kinetics and levels of model variables to the generalized picture of the HBV lifecycle based on the information available in the cited literature.

Overall, in this study, we examined (1) novel targets for antiviral therapy and (2) the efficacy of combination therapies with approved and/or clinically evaluated drugs. The differences in HBV strains can be accommodated in the model via parameter refinement subject to the availability of relevant virological data. The HBV lifecycle model can be employed as a module for multiscale integration with the ‘in-host’ and ‘between-host’ population dynamics of infection.

## Figures and Tables

**Table 1:** Summary of the existing models of HBV replication [12,13,14,15,16,17,18].

Model	Number of Variables	TypeofModel	ModelingStudyObjectives
[12]	9	ODEs	Switching between replication patterns during within-host evolution of HBV
[13]	11	ODEs	Effect of virion recycling on switching between intracellular replication patterns
[14]	7	Multiscale, age-dependent PDEs/ODEs	Effect of capsid assembly modulators on chronic HBV infection
[15]	5	Multiscale, age-dependent PDEs/ODEs	Effect of capsid assembly modulators and nucleoside analogues
[16]	8	Multiscale model, ODEs	Analysis of reproduction number, transmission dynamics, stability and sensitivity
[17]	7	ODEs + discrete agent-based stochastic MC	Effect of refractory cell emergence on viremia clearance and hepatocyte turnover
[18]	329	Discrete stochastic MC (detailed description of encapsidation and SVP formation)	Prediction and comparison of various treatment strategies

**Table 2:** Time-dependent variables of the HBV lifecycle model and their biochemical meaning.

#	Variable	Meaning
1	$\left[V_{\mathrm{free}}\right]$	free complete virions (rcDNA + HBs + HBc) outside the cell membrane
2	$\left[V_{\mathrm{bound}}\right]$	complete virions bound to α-DG receptor
3	$\left[V_{\mathrm{endosome}}\right]$	complete virions in endosomes
4	$\left[{\mathrm{rcDNA}}_{\mathrm{cor}}^{cyt}\right]$	capsids with partially double-stranded relaxed circular DNA (rcDNA) in cytoplasm
5	$\left[{\mathrm{rcDNA}}_{\mathrm{cor}}^{nuc}\right]$	capsids with partially double-stranded relaxed circular DNA (rcDNA) in nucleoplasm
6	$\left[{\mathrm{cccDNA}}^{nuc}\right]$	covalently closed circular DNA (cccDNA) in nucleoplasm
7	$\left[{\mathrm{pgRNA}}^{nuc}\right]$	viral pregenomic RNA for the core (HBc) and polymerase (Pol) (reverse transcriptase) in the nucleoplasm
8	$\left[{\mathrm{mRNA}}_{\mathrm{preC}}^{nuc}\right]$	viral RNA for the precore protein in the nucleoplasm
9	$\left[{\mathrm{mRNA}}_{\mathrm{preS}1}^{nuc}\right]$	viral RNA for the L envelope protein in the nucleoplasm
10	$\left[{\mathrm{mRNA}}_{\mathrm{preS}2/S}^{nuc}\right]$	viral RNA for the M and S envelope protein in the nucleoplasm
11	$\left[{\mathrm{mRNA}}_{X}^{nuc}\right]$	viral RNA for the X protein in the nucleoplasm
12	$\left[{\mathrm{pgRNA}}^{cyt}\right]$	viral pregenomic RNA for the HBc and Pol in the cytoplasm
13	$\left[{\mathrm{mRNA}}_{\mathrm{preC}}^{cyt}\right]$	viral RNA for the HBe protein in the cytoplasm
14	$\left[{\mathrm{mRNA}}_{\mathrm{preS}1}^{cyt}\right]$	viral RNA for the L envelope protein in the cytoplasm
15	$\left[{\mathrm{mRNA}}_{\mathrm{preS}2/S}^{cyt}\right]$	viral RNA for the M and S envelope proteins in the cytoplasm
16	$\left[{\mathrm{mRNA}}_{X}^{cyt}\right]$	viral RNA for the X protein in the cytoplasm
17	[HBc]	HBc monomers translated from pgRNA
18	[Pol]	Pol proteins translated from pgRNA
19	[L-HBs]	L-HBs proteins translated from preS1 mRNA
20	[M-,S-HBS]	M-HBs and S-HBs proteins translated from preS2/S mRNA
21	[HBe]	HBe proteins translated from precore mRNA and dimerized
22	[HBx]	X regulatory proteins translated from X mRNA
23	$\left[{\mathrm{CAP}}_{\mathrm{pgRNA}}\right]$	capsids assembled from pgRNA, HBc and Pol proteins in the cytoplasm
24	$\left[{\mathrm{CAP}}_{(-)\mathrm{DNA}}\right]$	capsids with the (−)DNA and the 5’-terminal RNA fragment (following (−) strand synthesis stage of reverse transcription)
25	$\left[{\mathrm{CAP}}_{\mathrm{rcDNA}}\right]$	capsids with relaxed circular partially double-stranded DNA (following (+) strand synthesis stage of reverse transcription)
26	$\left[{\mathrm{CAP}}_{\mathrm{empty}}\right]$	empty capsids without pgRNA assembled from HBc proteins in the cytoplasm
27	$\left[V_{\mathrm{ass}}\right]$	assembled complete viral particles at the endoplasmic reticulum
28	$\left[V_{\sec}\right]$	complete virions (Dane particles) secreted out of the cell
29	$\left[V_{\mathrm{ass}}^{\mathrm{HBs}}\right]$	assembled subviral particles (HBs spheres/filaments) in the cytoplasm
30	$\left[V_{\mathrm{ass}}^{\mathrm{empty}}\right]$	assembled empty virions (enveloped empty capsids)
31	$\left[V_{\sec}^{\mathrm{HBs}}\right]$	free subviral particles (HBs spheres/filaments) secreted out of the cell
32	$\left[V_{\sec}^{\mathrm{empty}}\right]$	empty virions (enveloped empty capsids) secreted out of the cell
33	[HBeAg]	free HBeAg particles secreted out of the cell

**Table 3:** Description of the HBV model parameters.

Parameter	Description,Units	Value,AdmissibleRange	Refs.
$k_{\mathrm{bind}}$	rate of virion binding to NTCP receptor, $h^{-1}$	6.84, [3, 70]	[33]
$d_{V}$	degradation rate of free extracellular virions, $h^{-1}$	0.17, [0.16, 0.18]	[17,32]
$k_{\mathrm{diss}}$	dissociation rate constant of bound virions, $h^{-1}$	209, [182, 236]	[33]
$k_{\mathrm{fuse}}$	fusion and entry by endocytosis rate, $h^{-1}$	1	calibration, [34,42]
$k_{\mathrm{uncoat}}$	uncoating rate constant, $h^{-1}$	0.8	calibration, [18,34,42]
$d_{\mathrm{endosome}}$	degradation rate of virions in endosomes, $h^{-1}$	0.02	assumed to be $\approx \;0.1\;d_{V}$
$k_{\mathrm{trp}}$	transport rate of rcDNA to nucleus, $h^{-1}$	0.08	calibration, [18,34,42]
$d_{\mathrm{rcDNA}}$	degradation rate of rcDNA in cytoplasm/nucleus, $h^{-1}$	0.0006 (0.0005, 0.0007)	assumed to be $=\;d_{\mathrm{cccDNA}}$ as in [18]
$k_{\mathrm{rep}}$	conversion rate of rcDNA to cccDNA, $h^{-1}$	0.08	calibration, [34]
$d_{\mathrm{cccDNA}}$	degradation rate of cccDNA in nucleus, $h^{-1}$	0.0006 (0.0005, 0.0009)	[3,18,20,37]
$k_{\mathrm{trnsc}}$	active transcription rate from cccDNA, nt · h^−1^	90,000	[18,46]
$\varepsilon_{\mathrm{HBx}}$	factor for (silenced) transcription rate in the absence of HBx	0.3	calibration, [25,51,52]
$K_{\mathrm{HBx}}$	threshold for cytoplasmic HBx level needed for active transcription from cccDNA, molecules	$10^{4}$	calibration, [25]
$\ell_{\mathrm{pgRNA}}$	length of HBV genome coding pgRNA, nt	3500	[11]
$\ell_{\mathrm{preC}}$	length of HBV genome coding precore mRNA, nt	3500	[11]
$\ell_{\mathrm{preS}1}$	length of HBV genome coding preS1 mRNA, nt	2400	[11]
$\ell_{\mathrm{preS}2/S}$	length of HBV genome coding preS2/S mRNA, nt	2100	[11]
$\ell_{\mathrm{mX}}$	length of HBV genome coding X mRNA, nt	700	[11]
$d_{\mathrm{pgRNA}}$	degradation rate of pgRNA, $h^{-1}$	0.13 [0.06, 0.14]	[18,48,57,58]
$d_{\mathrm{preC}}^{\mathrm{mRNA}}$	degradation rate of precore mRNA, $h^{-1}$	0.13 [0.09, 0.14]	[18,48,57]
$d_{\mathrm{preS}1}^{\mathrm{mRNA}}$	degradation rate of preS1 mRNA, $h^{-1}$	0.18 [0.17, 0.23]	[18,42]
$d_{\mathrm{preS}2/S}^{\mathrm{mRNA}}$	degradation rate of preS2/S mRNA, $h^{-1}$	0.18 [0.17, 0.23]	[18,42]
$d_{{\mathrm{mRNA}}_{X}}$	degradation rate of X mRNA, $h^{-1}$	0.21 [0.2, 0.22]	[49]
$k_{\mathrm{tp}}$	nuclear export rate of RNA molecules, $h^{-1}$	2.3 [2.1, 4.6]	[53]
$k_{\mathrm{trans}}$	protein translation rate, aa/h	12,000	[18,47]
$\ell_{\mathrm{HBc}}$	length of pgRNA translated into HBc protein, aa	183	[11]
$\ell_{\mathrm{Pol}}$	length of pgRNA translated into viral polymerase (Pol) protein, aa	845	[11]
$\ell_{\mathrm{HBe}}$	length of precore mRNA translated into HBe protein, aa	159	[11]
$\ell_{\mathrm{HBs}}$	length of preS1 mRNA translated into L-HBs protein, aa	400	[11]
$\ell_{S}$	length of preS2/S mRNA translated into M/S-HBs, aa	254 [226, 281]	[11]
$\ell_{\mathrm{HBx}}$	length of X mRNA translated into HBx protein, aa	154	[11]
$d_{\mathrm{HBc}}$	degradation rate of core protein, $h^{-1}$	0.69	[18]
$d_{\mathrm{Pol}}$	degradation rate of Pol protein, $h^{-1}$	0.69	[18]
$d_{\mathrm{HBe}}$	degradation rate of HBe protein, $h^{-1}$	0.69	[18]
$d_{L\text{-}\mathrm{HBs}}$	degradation rate of L-HBs protein, $h^{-1}$	0.69	[18]
$d_{M\text{-},S\text{-}\mathrm{HBs}}$	degradation rate of M/S-HBs protein, $h^{-1}$	0.69	[18]
$d_{\mathrm{HBx}}$	degradation rate of HBx protein, $h^{-1}$	0.69	[18]
$k_{(-)\mathrm{RT}}$	rate of (−) strand DNA synthesis, $h^{-1}$	0.029	[18]
$k_{(+)\mathrm{RT}}$	rate of (+) strand DNA synthesis, $h^{-1}$	0.058	[18]
$k_{\mathrm{cap}}$	nucleocapsid assembly rate constant per pgRNA, h^−1^	1.2	calibration, [14,18]
$k_{\mathrm{cap}}^{\mathrm{empty}}$	empty capsid assembly rate constant, h^−1^	600	$=\;500\;k_{\mathrm{cap}}$, calibration
$K_{\mathrm{cap}}$	threshold number of capsids for requirements on HBc/Pol availability	1000	[18]
$N_{\mathrm{HBc}}$	number of core proteins per capsid, molecules	120	[18]
$N_{\mathrm{Pol}}$	number of polymerase proteins per capsid, molecules	1	[18]
$N_{L\text{-}\mathrm{HBs}}$	number of L-HBs proteins per complete or empty virion, molecules	20	[18]
$N_{M\text{-},S\text{-}\mathrm{HBs}}$	number of M-,S-HBs proteins per complete or empty virion, molecules	80	[18]
$N_{L\text{-}\mathrm{HBs}}^{\mathrm{SVP}}$	number of L-HBs proteins per subviral particle, molecules	10	[18]
$N_{M\text{-},S\text{-}\mathrm{HBs}}^{\mathrm{SVP}}$	number of M-,S-HBS proteins per subviral particle, molecules	50	[18]
$d_{\mathrm{pgRNA}}^{\mathrm{CAP}}$	degradation rate of pgRNA-containing capsids, $h^{-1}$	0.08 [0.03, 0.1]	[18,58]
$d_{(-)\mathrm{DNA}}^{\mathrm{CAP}}$	degradation rate of (-)DNA-containing capsids, $h^{-1}$	0.08 [0.03, 0.1]	[18,58]
$d_{\mathrm{rcDNA}}^{\mathrm{CAP}}$	degradation rate of rcDNA-containing capsids, $h^{-1}$	0.08 [0.03, 0.1]	[18,58]
$d_{{\mathrm{CAP}}_{\mathrm{empty}}}$	degradation rate of empty capsids, $h^{-1}$	0.08 [0.03, 0.1]	[18,58]
$k_{\mathrm{intrec}}$	intracellular recycling rate of rcDNA-containing capsids, $h^{-1}$	$5\;\cdot \;10^{-4}$	calibration, [20,22,25]
$k_{\mathrm{ass}}^{V_{\mathrm{ass}}}$	assembly rate of complete virions per nucleocapsid, $h^{-1}$	1.2	calibration
$k_{\sec}^{V_{\mathrm{ass}}}$	secretion rate of infectious virions (Dane particles), $h^{-1}$	0.3	[17,18,32]
$k_{\mathrm{ass}}^{V_{\mathrm{empty}}}$	assembly rate of empty virions per empty capsid, $h^{-1}$	8	calibration
$k_{\sec}^{V_{\mathrm{empty}}}$	secretion rate of empty virions (enveloped empty capsids), $h^{-1}$	0.3	$=\;k_{\sec}^{V_{\mathrm{ass}}}$, [18,21]
$k_{\mathrm{ass}}^{\mathrm{SVP}}$	assembly rate of subviral particles (HBs spheres/filaments), $h^{-1}$	$8\;\cdot \;10^{4}$	calibration
$k_{\sec}^{\mathrm{SVP}}$	secretion rate of subviral particles (HBs spheres/filaments), $h^{-1}$	0.3	$=\;k_{\sec}^{V_{\mathrm{ass}}}$, [18,21]
$k_{\sec}^{\mathrm{HBe}}$	secretion rate of HBeAg particles, $h^{-1}$	4 [3, 5]	[40,41,42]
$d_{V_{\mathrm{HBs}}}$	degradation rate of secreted HBsAg particles (SVP or empty virions), $h^{-1}$	0.17 [0.16, 0.18]	$=\;d_{V}$, [17]
$d_{\mathrm{HBeAg}}$	degradation rate of secreted HBeAg particles, $h^{-1}$	0.69	[18]

**Table 4:** The Gillespie-based stochastic model of HBV replication. The set of elementary reactions implemented by Markov chain are presented. Superscripts “ ^−−^” and “^++^” denote, respectively, the decrease and increase in population of the corresponding component by one particle during the transition.

*m*	Transition	Propensity	*m*	Transition	Propensity
1	${\left[V_{\mathrm{free}}\right]}^{--},{\left[V_{\mathrm{bound}}\right]}^{++}$	$k_{\mathrm{bind}}\left[V_{\mathrm{free}}\right]$	38	${\left[\mathrm{HBc}\right]}^{--}$	$d_{\mathrm{HBc}}\left[\mathrm{HBc}\right]$
2	${\left[V_{\mathrm{free}}\right]}^{++},{\left[V_{\mathrm{bound}}\right]}^{--}$	$k_{\mathrm{diss}}\left[V_{\mathrm{bound}}\right]$	39	${\left[\mathrm{Pol}\right]}^{++}$	$k_{\mathrm{trans}}f_{\mathrm{Pol}}\left[{\mathrm{pgRNA}}^{cyt}\right]$
3	${\left[V_{\mathrm{free}}\right]}^{--}$	$d_{V}\left[V_{\mathrm{free}}\right]$	40	${\left[\mathrm{Pol}\right]}^{--}$	$k_{\mathrm{cap}}F_{\mathrm{cap}}N_{\mathrm{Pol}}\left[{\mathrm{pgRNA}}^{cyt}\right]$
4	${\left[V_{\mathrm{bound}}\right]}^{--},{\left[V_{\mathrm{endosome}}\right]}^{++}$	$k_{\mathrm{fuse}}\left[V_{\mathrm{bound}}\right]$	41	${\left[\mathrm{Pol}\right]}^{--}$	$d_{\mathrm{Pol}}\left[\mathrm{Pol}\right]$
5	${\left[V_{\mathrm{bound}}\right]}^{--}$	$d_{V}\left[V_{\mathrm{bound}}\right]$	42	${\left[L\text{-}\mathrm{HBs}\right]}^{++}$	$k_{\mathrm{trans}}f_{\mathrm{LHBs}}\left[{\mathrm{mRNA}}_{\mathrm{preS}1}^{cyt}\right]$
6	${\left[V_{\mathrm{endosome}}\right]}^{--},{\left[{\mathrm{rcDNA}}_{\mathrm{cor}}^{cyt}\right]}^{++}$	$k_{\mathrm{uncoat}}\left[V_{\mathrm{endosome}}\right]$	43	${\left[L\text{-}\mathrm{HBs}\right]}^{--}$	$N_{\mathrm{LHBs}}k_{\mathrm{ass}}^{\mathrm{Vass}}F_{\mathrm{ass}}\left[{\mathrm{CAP}}_{\mathrm{rcDNA}}\right]$
7	${\left[V_{\mathrm{endosome}}\right]}^{--}$	$d_{\mathrm{endosome}}\left[V_{\mathrm{endosome}}\right]$	44	${\left[L\text{-}\mathrm{HBs}\right]}^{--}$	$N_{\mathrm{LHBs}}k_{\mathrm{ass}}^{\mathrm{Vempty}}F_{\mathrm{ass}}\left[{\mathrm{CAP}}_{\mathrm{empty}}\right]$
8	${\left[{\mathrm{rcDNA}}_{\mathrm{cor}}^{cyt}\right]}^{--},{\left[{\mathrm{rcDNA}}_{\mathrm{cor}}^{nuc}\right]}^{++}$	$k_{\mathrm{trp}}\left[{\mathrm{rcDNA}}_{\mathrm{cor}}^{cyt}\right]$	45	${\left[L\text{-}\mathrm{HBs}\right]}^{--}$	$k_{\mathrm{ass}}^{\mathrm{SVP}}N_{\mathrm{SVP}}^{\mathrm{LHBs}}F_{\mathrm{ass}}$
9	${\left[{\mathrm{rcDNA}}_{\mathrm{cor}}^{cyt}\right]}^{--}$	$d_{\mathrm{rcDNA}}\left[{\mathrm{rcDNA}}_{\mathrm{cor}}^{cyt}\right]$	46	${\left[L\text{-}\mathrm{HBs}\right]}^{--}$	$d_{\mathrm{LHBs}}\left[L\text{-}\mathrm{HBs}\right]$
10	${\left[{\mathrm{rcDNA}}_{\mathrm{cor}}^{nuc}\right]}^{--},{\left[{\mathrm{cccDNA}}^{nuc}\right]}^{++}$	$k_{\mathrm{rep}}\left[{\mathrm{rcDNA}}_{\mathrm{cor}}^{nuc}\right]$	47	${\left[M\text{-},S\text{-}\mathrm{HBs}\right]}^{++}$	$k_{\mathrm{trans}}f_{\mathrm{MSHBs}}\left[{\mathrm{mRNA}}_{\mathrm{preS}2/S}^{cyt}\right]$
11	${\left[{\mathrm{rcDNA}}_{\mathrm{cor}}^{nuc}\right]}^{--}$	$d_{\mathrm{rcDNA}}\left[{\mathrm{rcDNA}}_{\mathrm{cor}}^{nuc}\right]$	48	${\left[M\text{-},S\text{-}\mathrm{HBs}\right]}^{--}$	$N_{\mathrm{MSHBs}}k_{\mathrm{ass}}^{\mathrm{Vass}}F_{\mathrm{ass}}\left[{\mathrm{CAP}}_{\mathrm{rcDNA}}\right]$
12	${\left[{\mathrm{rcDNA}}_{\mathrm{cor}}^{nuc}\right]}^{++},{\left[{\mathrm{CAP}}_{\mathrm{rcDNA}}\right]}^{--}$	$k_{\mathrm{intrec}}\left[{\mathrm{CAP}}_{\mathrm{rcDNA}}\right]$	49	${\left[M\text{-},S\text{-}\mathrm{HBs}\right]}^{--}$	$N_{\mathrm{MSHBs}}k_{\mathrm{ass}}^{\mathrm{Vempty}}F_{\mathrm{ass}}\left[{\mathrm{CAP}}_{\mathrm{empty}}\right]$
13	${\left[{\mathrm{cccDNA}}^{nuc}\right]}^{--}$	$d_{\mathrm{cccDNA}}\left[{\mathrm{cccDNA}}^{nuc}\right]$	50	${\left[M\text{-},S\text{-}\mathrm{HBs}\right]}^{--}$	$k_{\mathrm{ass}}^{\mathrm{SVP}}N_{\mathrm{MSHBs}}^{\mathrm{SVP}}F_{\mathrm{ass}}$
14	${\left[{\mathrm{pgRNA}}^{nuc}\right]}^{++}$	$k_{\mathrm{trnsc}}l_{\mathrm{pgRNA}}^{-1}\theta_{\mathrm{HBx}}\left[{\mathrm{cccDNA}}^{nuc}\right]$	51	${\left[M\text{-},S\text{-}\mathrm{HBs}\right]}^{--}$	$d_{M\text{-},S\text{-}\mathrm{HBs}}\left[M\text{-},S\text{-}\mathrm{HBs}\right]$
15	${\left[{\mathrm{pgRNA}}^{nuc}\right]}^{--},{\left[{\mathrm{pgRNA}}^{cyt}\right]}^{++}$	$k_{\mathrm{tp}}\left[{\mathrm{pgRNA}}^{nuc}\right]$	52	${\left[\mathrm{HBe}\right]}^{++}$	$k_{\mathrm{trans}}f_{\mathrm{HBe}}\left[{\mathrm{mRNA}}_{\mathrm{preC}}^{cyt}\right]$
16	${\left[{\mathrm{pgRNA}}^{nuc}\right]}^{--}$	$d_{\mathrm{pgRNA}}\left[{\mathrm{pgRNA}}^{nuc}\right]$	53	${\left[\mathrm{HBe}\right]}^{--},{\left[\mathrm{HBeAg}\right]}^{++}$	$k_{\sec}^{\mathrm{HBe}}\left[\mathrm{HBe}\right]$
17	${\left[{\mathrm{mRNA}}_{\mathrm{preC}}^{nuc}\right]}^{++}$	$k_{\mathrm{trnsc}}l_{\mathrm{pgRNA}}^{-1}\theta_{\mathrm{HBx}}\left[{\mathrm{cccDNA}}^{nuc}\right]$	54	${\left[\mathrm{HBe}\right]}^{--}$	$d_{\mathrm{HBe}}\left[\mathrm{HBe}\right]$
18	${\left[{\mathrm{mRNA}}_{\mathrm{preC}}^{nuc}\right]}^{--},{\left[{\mathrm{mRNA}}_{\mathrm{preC}}^{cyt}\right]}^{++}$	$k_{\mathrm{tp}}\left[{\mathrm{mRNA}}_{\mathrm{preC}}^{nuc}\right]$	55	${\left[\mathrm{HBx}\right]}^{++}$	$1.0\times k_{\mathrm{trans}}f_{\mathrm{HBx}}\left[{\mathrm{mRNA}}_{X}^{cyt}\right]$
19	${\left[{\mathrm{mRNA}}_{\mathrm{preC}}^{nuc}\right]}^{--}$	$d_{\mathrm{preC}}^{\mathrm{mRNA}}\left[{\mathrm{mRNA}}_{\mathrm{preC}}^{nuc}\right]$	56	${\left[\mathrm{HBx}\right]}^{--}$	$d_{\mathrm{HBx}}\left[\mathrm{HBx}\right]$
20	${\left[{\mathrm{mRNA}}_{\mathrm{preS}1}^{nuc}\right]}^{++}$	$k_{\mathrm{trnsc}}l_{\mathrm{preS}1}^{-1}\theta_{\mathrm{HBx}}\left[{\mathrm{cccDNA}}^{nuc}\right]$	57	${\left[{\mathrm{CAP}}_{\mathrm{pgRNA}}\right]}^{--},{\left[{\mathrm{CAP}}_{(-)\mathrm{DNA}}\right]}^{++}$	$k_{(-)\mathrm{RT}}\left[{\mathrm{CAP}}_{\mathrm{pgRNA}}\right]$
21	${\left[{\mathrm{mRNA}}_{\mathrm{preS}1}^{nuc}\right]}^{--},{\left[{\mathrm{mRNA}}_{\mathrm{preS}1}^{cyt}\right]}^{++}$	$k_{\mathrm{tp}}\left[{\mathrm{mRNA}}_{\mathrm{preS}1}^{nuc}\right]$	58	${\left[{\mathrm{CAP}}_{\mathrm{pgRNA}}\right]}^{--}$	$d_{\mathrm{CAP}}\left[{\mathrm{CAP}}_{\mathrm{pgRNA}}\right]$
22	${\left[{\mathrm{mRNA}}_{\mathrm{preS}1}^{nuc}\right]}^{--}$	$d_{\mathrm{preS}1}^{\mathrm{mRNA}}\left[{\mathrm{mRNA}}_{\mathrm{preS}1}^{nuc}\right]$	59	${\left[{\mathrm{CAP}}_{(-)\mathrm{DNA}}\right]}^{--},{\left[{\mathrm{CAP}}_{\mathrm{rcDNA}}\right]}^{++}$	$k_{(+)\mathrm{RT}}\left[{\mathrm{CAP}}_{(-)\mathrm{DNA}}\right]$
23	${\left[{\mathrm{mRNA}}_{\mathrm{preS}2/S}^{nuc}\right]}^{++}$	$k_{\mathrm{trnsc}}l_{\mathrm{preS}2/S}^{-1}\theta_{\mathrm{HBx}}\left[{\mathrm{cccDNA}}^{nuc}\right]$	60	${\left[{\mathrm{CAP}}_{(-)\mathrm{DNA}}\right]}^{--}$	$d_{\mathrm{CAP}}\left[{\mathrm{CAP}}_{(-)\mathrm{DNA}}\right]$
24	${\left[{\mathrm{mRNA}}_{\mathrm{preS}2/S}^{nuc}\right]}^{--},{\left[{\mathrm{mRNA}}_{\mathrm{preS}2/S}^{cyt}\right]}^{++}$	$k_{\mathrm{tp}}\left[{\mathrm{mRNA}}_{\mathrm{preS}2/S}^{nuc}\right]$	61	${\left[{\mathrm{CAP}}_{\mathrm{rcDNA}}\right]}^{--},{\left[V_{\mathrm{ass}}\right]}^{++}$	$k_{\mathrm{ass}}^{\mathrm{Vass}}F_{\mathrm{ass}}\left[{\mathrm{CAP}}_{\mathrm{rcDNA}}\right]$
25	${\left[{\mathrm{mRNA}}_{\mathrm{preS}2/S}^{nuc}\right]}^{--}$	$d_{\mathrm{preS}2/S}^{\mathrm{mRNA}}\left[{\mathrm{mRNA}}_{\mathrm{preS}2/S}^{nuc}\right]$	62	${\left[{\mathrm{CAP}}_{\mathrm{rcDNA}}\right]}^{--}$	$d_{\mathrm{CAP}}\left[{\mathrm{CAP}}_{\mathrm{rcDNA}}\right]$
26	${\left[{\mathrm{mRNA}}_{X}^{nuc}\right]}^{++}$	$k_{\mathrm{trnsc}}l_{\mathrm{mX}}^{-1}\theta_{\mathrm{HBx}}\left[{\mathrm{cccDNA}}^{nuc}\right]$	63	${\left[{\mathrm{CAP}}_{\mathrm{empty}}\right]}^{++}$	$k_{\mathrm{cap}}^{\mathrm{empty}}F_{\mathrm{cap}}^{\mathrm{empty}}$
27	${\left[{\mathrm{mRNA}}_{X}^{nuc}\right]}^{--},{\left[{\mathrm{mRNA}}_{X}^{cyt}\right]}^{++}$	$k_{\mathrm{tp}}\left[{\mathrm{mRNA}}_{X}^{nuc}\right]$	64	${\left[{\mathrm{CAP}}_{\mathrm{empty}}\right]}^{--},{\left[V_{\mathrm{ass}}^{\mathrm{empty}}\right]}^{++}$	$k_{\mathrm{ass}}^{\mathrm{Vempty}}F_{\mathrm{ass}}\left[{\mathrm{CAP}}_{\mathrm{empty}}\right]$
28	${\left[{\mathrm{mRNA}}_{X}^{nuc}\right]}^{--}$	$d_{X}^{\mathrm{mRNA}}\left[{\mathrm{mRNA}}_{X}^{nuc}\right]$	65	${\left[{\mathrm{CAP}}_{\mathrm{empty}}\right]}^{--}$	$d_{\mathrm{CAP}}\left[{\mathrm{CAP}}_{\mathrm{empty}}\right]$
29	${\left[{\mathrm{pgRNA}}^{cyt}\right]}^{--},{\left[{\mathrm{CAP}}_{\mathrm{pgRNA}}\right]}^{++}$	$k_{\mathrm{cap}}F_{c}ap\left[{\mathrm{pgRNA}}^{cyt}\right]$	66	${\left[V_{\mathrm{ass}}\right]}^{--},{\left[V_{\sec}\right]}^{++},{\left[V_{\mathrm{cum}}\right]}^{++}$	$k_{\sec}^{\mathrm{Vass}}\left[V_{\mathrm{ass}}\right]$
30	${\left[{\mathrm{pgRNA}}^{cyt}\right]}^{--}$	$d_{\mathrm{pgRNA}}\left[{\mathrm{pgRNA}}^{cyt}\right]$	67	${\left[V_{\sec}\right]}^{--}$	$d_{V}\left[V_{\sec}\right]$
31	${\left[{\mathrm{mRNA}}_{\mathrm{preC}}^{cyt}\right]}^{--}$	$d_{\mathrm{preC}}^{\mathrm{mRNA}}\left[{\mathrm{mRNA}}_{\mathrm{preC}}^{cyt}\right]$	68	${\left[V_{\mathrm{ass}}^{\mathrm{HBs}}\right]}^{++}$	$k_{\mathrm{ass}}^{\mathrm{SVP}}F_{\mathrm{ass}}$
32	${\left[{\mathrm{mRNA}}_{\mathrm{preS}1}^{cyt}\right]}^{--}$	$d_{\mathrm{preS}1}^{\mathrm{mRNA}}\left[{\mathrm{mRNA}}_{\mathrm{preS}1}^{cyt}\right]$	69	${\left[V_{\mathrm{ass}}^{\mathrm{HBs}}\right]}^{--},{\left[V_{\sec}^{\mathrm{HBs}}\right]}^{++}$	$k_{\sec}^{\mathrm{SVP}}\left[V_{\mathrm{ass}}^{\mathrm{HBs}}\right]$
33	${\left[{\mathrm{mRNA}}_{\mathrm{preS}2/S}^{cyt}\right]}^{--}$	$d_{\mathrm{preS}2/S}^{\mathrm{mRNA}}\left[{\mathrm{mRNA}}_{\mathrm{preS}2/S}^{cyt}\right]$	70	${\left[V_{\mathrm{ass}}^{\mathrm{empty}}\right]}^{--},{\left[V_{\sec}^{\mathrm{empty}}\right]}^{++}$	$k_{\sec}^{\mathrm{Vempty}}\left[V_{\mathrm{ass}}^{\mathrm{empty}}\right]$
34	${\left[{\mathrm{mRNA}}_{X}^{cyt}\right]}^{--}$	$d_{X}^{\mathrm{mRNA}}\left[{\mathrm{mRNA}}_{X}^{cyt}\right]$	71	${\left[V_{\sec}^{\mathrm{HBs}}\right]}^{--}$	$d_{V_{H}\mathrm{Bs}}\left[V_{\sec}^{\mathrm{HBs}}\right]$
35	${\left[\mathrm{HBc}\right]}^{++}$	$k_{\mathrm{trans}}f_{\mathrm{HBc}}\left[{\mathrm{pgRNA}}^{cyt}\right]$	72	${\left[V_{\sec}^{\mathrm{empty}}\right]}^{--}$	$d_{V_{\mathrm{HBs}}}\left[V_{\sec}^{\mathrm{empty}}\right]$
36	${\left[\mathrm{HBc}\right]}^{--}$	$N_{\mathrm{HBc}}k_{\mathrm{cap}}F_{\mathrm{cap}}\left[{\mathrm{pgRNA}}^{cyt}\right]$	73	${\left[\mathrm{HBeAg}\right]}^{--}$	$d_{\mathrm{HBeAg}}\left[\mathrm{HBeAg}\right]$
37	${\left[\mathrm{HBc}\right]}^{--}$	$N_{\mathrm{HBc}}k_{\mathrm{cap}}^{\mathrm{empty}}F_{\mathrm{cap}}^{\mathrm{empty}}$			

**Table 5:** Parameter sensitivity indices $S_{k}$ calculated for MOI=20.

Parameter	$S_{k}$	Parameter	$S_{k}$	Parameter	$S_{k}$	Parameter	$S_{k}$
$d_{\mathrm{CAP}}$	−3.66	$k_{\mathrm{ass}}^{\mathrm{Vass}}$	0.80	$K_{\mathrm{HBx}}$	−0.12	$l_{L\text{-}\mathrm{HBs}}$	−0.014
$k_{\mathrm{trnsc}}$	3.46	$k_{\mathrm{ass}}^{\mathrm{SVP}}$	−0.76	$d_{X}^{\mathrm{mRNA}}$	−0.12	$l_{\mathrm{preS}1}$	−0.014
$l_{\mathrm{pgRNA}}$	−2.50	$N_{M\text{-},S\text{-}\mathrm{HBs}}^{\mathrm{SVP}}$	−0.75	$d_{\mathrm{HBx}}$	−0.11	$N_{L-\mathrm{HBs}}$	−0.009
$k_{\mathrm{fuse}}$	2.18	$N_{\mathrm{HBc}}$	−0.68	$l_{\mathrm{Pol}}$	−0.084	$d_{L\text{-}\mathrm{HBs}}$	−0.009
$d_{V}$	−2.12	$l_{\mathrm{HBc}}$	−0.68	$N_{\mathrm{Pol}}$	−0.084	$N_{\mathrm{LHBs}}^{\mathrm{SVP}}$	−0.005
$k_{\mathrm{bind}}$	2.12	$k_{\mathrm{cap}}$	0.63	$d_{\mathrm{Pol}}$	−0.084	$l_{\mathrm{preC}}$	$10^{-12}$
$k_{\mathrm{diss}}$	−2.11	$K_{\mathrm{cap}}$	−0.42	$k_{\mathrm{uncoat}}$	0.079	$l_{\mathrm{HBe}}$	$-10^{-12}$
$k_{\mathrm{trp}}$	1.72	$k_{\mathrm{rep}}$	0.36	$N_{M\text{-},S\text{-}\mathrm{HBs}}$	−0.074	$d_{\mathrm{HBe}}$	$10^{-13}$
$d_{\mathrm{pgRNA}}$	−1.44	$d_{\mathrm{HBc}}$	−0.30	$d_{\mathrm{endosome}}$	−0.062	$d_{\mathrm{HBeAg}}$	$-10^{-13}$
$k_{(-)\mathrm{RT}}$	1.43	$k_{\mathrm{cap}}^{\mathrm{empty}}$	−0.29	$\varepsilon_{\mathrm{HBx}}$	0.059	$d_{\mathrm{preC}}^{\mathrm{mRNA}}$	$10^{-13}$
$k_{(+)\mathrm{RT}}$	1.15	$k_{\mathrm{tp}}$	0.22	$k_{\sec}^{Vass}$	0.041	$k_{\sec}^{\mathrm{HBe}}$	$10^{-13}$
$k_{\mathrm{intrec}}$	0.87	$k_{\mathrm{trp}}$	0.20	$d_{\mathrm{rcDNA}}$	−0.041	$k_{\sec}^{\mathrm{Vempty}}$	0
$d_{\mathrm{preS}2/S}^{\mathrm{mRNA}}$	−0.86	$d_{\mathrm{cccDNA}}$	−0.16	$k_{\mathrm{ass}}^{\mathrm{Vempty}}$	−0.026	$k_{\sec}^{\mathrm{SVP}}$	0
$l_{\mathrm{preS}2/S}$	−0.82	$l_{\mathrm{mX}}$	−0.12	$d_{M\text{-},S\text{-}\mathrm{HBs}}$	−0.019	$d_{V_{\mathrm{HBs}}}$	0
$l_{M\text{-},S\text{-}\mathrm{HBs}}$	−0.82	$l_{\mathrm{HBx}}$	−0.12	$d_{\mathrm{preS}1}^{\mathrm{mRNA}}$	−0.015		

## Data Availability

The original contributions presented in this study are included in the article. Further inquiries can be directed to the corresponding author(s).

## Abbreviations

The following abbreviations are used in this manuscript:

HBV	hepatitis B virus
rcDNA	relaxed circular DNA
cccDNA	covalently closed circular DNA
pgRNA	pregenomic RNA
Pol	polymerase
HBc	HBV core protein
HBe	HBV E protein
HBs	HBV surface (envelope) proteins (L, M and S)
WT	wild-type
SSA	stochastic simulation algorithm
ODE	ordinary differential equation
MC	Markov chain
